# Genome-wide analysis, expression profile of heat shock factor gene family (CaHsfs) and characterisation of CaHsfA2 in pepper (*Capsicum annuum* L.)

**DOI:** 10.1186/s12870-015-0512-7

**Published:** 2015-06-19

**Authors:** Meng Guo, Jin-Ping Lu, Yu-Fei Zhai, Wei-Guo Chai, Zhen-Hui Gong, Ming-Hui Lu

**Affiliations:** College of Horticulture, Northwest A&F University, Yangling, Shaanxi, 712100 P. R. China; Institute of Vegetables, Hangzhou Academy of Agricultural Sciences, Hangzhou, Zhejiang, 310024 P. R. China

**Keywords:** Pepper, Identification of CaHsfs family, Abiotic stress, CaHsfA2, Gene expression

## Abstract

**Background:**

Heat shock factors (Hsfs) play crucial roles in plant developmental and defence processes. The production and quality of pepper (*Capsicum annuum* L.), an economically important vegetable crop, are severely reduced by adverse environmental stress conditions, such as heat, salt and osmotic stress. Although the pepper genome has been fully sequenced, the characterization of the Hsf gene family under abiotic stress conditions remains incomplete.

**Results:**

A total of 25 CaHsf members were identified in the pepper genome by bioinformatics analysis and PCR assays. They were grouped into three classes, CaHsfA, B and C, based on highly conserved Hsf domains, were distributed over 11 of 12 chromosomes, with none found on chromosome 11, and all of them, except CaHsfA5, formed a protein–protein interaction network. According to the RNA-seq data of pepper cultivar CM334, most CaHsf members were expressed in at least one tissue among root, stem, leaf, pericarp and placenta. Quantitative real-time PCR assays showed that all of the CaHsfs responded to heat stress (40 °C for 2 h), except CaHsfC1 in thermotolerant line R9 leaves, and that the expression patterns were different from those in thermosensitive line B6. Many CaHsfs were also regulated by salt and osmotic stresses, as well as exogenous Ca^2+^, putrescine, abscisic acid and methyl jasmonate. Additionally, CaHsfA2 was located in the nucleus and had transcriptional activity, consistent with the typical features of Hsfs. Time-course expression profiling of CaHsfA2 in response to heat stress revealed differences in its expression level and pattern between the pepper thermosensitive line B6 and thermotolerant line R9.

**Conclusions:**

Twenty-five Hsf genes were identified in the pepper genome and most of them responded to heat, salt, osmotic stress, and exogenous substances, which provided potential clues for further analyses of CaHsfs functions in various kinds of abiotic stresses and of corresponding signal transduction pathways in pepper.

**Electronic supplementary material:**

The online version of this article (doi:10.1186/s12870-015-0512-7) contains supplementary material, which is available to authorized users.

## Background

Plants as sessile organisms have formed a variety of defence mechanisms to protect themselves from persistently changing stress factors, such as extreme temperature, salt and drought [1]. Temperature, especially high temperature, can affect crop growth and development, severely reducing the yield and quality [2–4]. Under heat stress (HS), the plant cells rapidly respond to a high temperature by inducing the expression of genes encoding heat shock proteins (Hsps), which are involved in preventing heat-related damage and confer plant thermotolerance [5, 6]. Many Hsps function as molecular chaperones in preventing protein misfolding and aggregation, consequently maintaining protein homeostasis in cells and causing the plant’s acquired thermotolerance [7–9].

Heat shock factors (*Hsfs*) regulate the expression of *Hsps* by recognizing heat shock elements (HSEs) within the promoters of Hsps [1, 10]. HSEs are characterised by multiple inverted repeats of the nGAAn sequence, and at least three HSE motifs are required for efficient Hsf oligomer binding in eukaryotic organisms [11, 12]. Under non-stress conditions, Hsfs are maintained in inactive states and form cytoplasmic complexes with Hsp90/Hsp70 chaperone complexes [8, 13]. Under HS conditions, as the result of a cytosolic protein response, Hsfs are released from chaperone complexes and bind to the HSEs of target genes after undergoing phosphorylation, sumoylation, trimerisation and nuclear import [1, 13, 14].

Hsf families share a conserved modular structure. Despite considerable variability in size and sequence, their structures and functions are conserved throughout the eukaryotic kingdom [8, 15]. In plant Hsfs, the highly conserved DNA-binding domain (DBD), which is composed of an antiparallel four-stranded *β*-sheet (*β*1, *β*2, *β*3 and *β*4) and three helical bundles (*α*1, *α*2 and *α*3) in the N-terminus is required for the positioning and recognition of HSEs [16–18]. The oligimerisation domain (OD or HR-A/B region), responsible for the transcription factor activity, is connected to the DBD by a flexible linker [1] and is composed of a heptad pattern of hydrophobic amino acid residues [19–21]. In addition, a cluster of basic amino acid residues, the nuclear localisation signal (NLS), essential for nuclear import, a leucine-rich nuclear export signal (NES) for nuclear export, short peptide motifs (AHA motifs) for activator functions, and a repressor domain (RD), characterised by the tetrapeptide LFGV in the C-terminus, exist in some Hsfs [1, 22–24].

The number of *Hsf* genes varies greatly among different eukaryotic organisms. *Drosophila melanogaster, Caenorhabditis elegans* and *Saccharomyces cerevisiae* have a single *Hsf* gene, and vertebrate genomes contain four *Hsf* genes [25]. In contrast to the low numbers of *Hsf* genes in animals and yeasts, plants possess large *Hsf* families, with 21 Hsf genes in Arabidopsis (*Arabidopsis thaliana*), 25 in rice (*Oryza sativa*), 30 in maize (*Zea mays*), 52 *Hsf* genes in soybean (*Glycine max),* and at least 24 in tomato (*Solanum lycopersicum*) [1], indicating that plant Hsfs in various species may have multiple functions in preventing stress damage [1, 26]. Based on the peculiarities of the HR-A/B regions, plant Hsfs are divided into three classes, A, B and C [20]. Class A and C Hsfs contain an extended HR-A/B with 21 and 7 amino acid residues between the HR-A and HR-B region, respectively, whereas class B Hsfs have a compact HR-A/B region lacking an insertion [15, 20]. Additionally, class A Hsfs contain aromatic (W, F, Y), hydrophobic (L, I, V) and acidic (D, E) AHA activation domains that are absent in class B and C Hsfs [24]. Class B Hsfs, except HsfB5, contain the RD in the C-terminus, which is speculated to function as a repressor motif, making HsfB members act as repressors [27–30]. However, *Arabidopsis* HsfB1 is able to positively regulate the acquired thermotolerance [29]. This apparent contradiction remains to be elucidated in future research.

Many plant Hsf genes from various species have been isolated and comprehensively studied. In *Arabidopsis*, HsfA1 and HsfA2 can synergistically activate target genes by forming superactivator heterodimers [31]. While negatively regulating the expression levels of heat-inducible *Hsfs*, HsfB1 and HsfB2b are necessary for acquired thermotolerance [29]. The expression of *HsfA9* increases during embryogenesis and seed maturation [32], whereas *HsfA5* is inactive and inhibits *HsfA4* activity in tomato [1]. HsfA2 can enhance the tolerance of plants to multiple abiotic stresses, such as HS [1], salt/osmotic stress [33], oxidative stress [34] and anoxia [35]. HsfA2 in tomato contributes to fruit set during HS by activating the protection mechanisms in the anther [26, 36].

Pepper (*Capsicum annuum* L.), a very important economic crop, is sensitive to HS; however, investigations regarding the molecular mechanisms of heat tolerance have been limited [37, 38]. The *Hsf* gene family has, so far, been fully characterised only in a few model species, such as *Arabidopsis*, rice, maize, wheat and Chinese cabbage [8, 20, 39–41]. The genome sequence of pepper has been published recently [42, 43], which enables the characterisation of the pepper *Hsf* family and their responses to various stresses at the molecular level. In this study, the genome-wide identification of the pepper *Hsf* family members is performed using bioinformatics and gene expression analyses. A total of 25 *Hsf* family members from pepper are identified using bioinformatics analysis and PCR tests. The gene structure, conserved domains, chromosomal location, gene duplication and phylogenetic analyses are presented. In addition, we analyze the expression patterns of *Hsf* genes in different pepper tissues, as well as their responses to various stresses. The results provide a foundation for further functional research on *Hsf* genes in pepper and will help to reveal the functions of *Hsf* genes in other plant species.

## Results

### Identification of the *Hsf* gene family in pepper

The Hidden Markov Model (HMM) profile of the Hsf DBD domain (Pfam: PF00447) (http://pfam.sanger.ac.uk/) was used as a BLAST query against the pepper genome database PGP (http://peppergenome.snu.ac.kr/), and the Hsf proteins in *Arabidopsis*, *Vitis vinifera* and *Populus trichocarpa* from the PTFD (Plant Transcription Factor Database, http://plntfdb.bio.uni-potsdam.de/v3.0/) were also used as BLAST query against PGP. A total of 26 candidate *Hsf* genes were originally obtained from pepper cultivar CM334, aligned with the corresponding genes in the cultivar Zunla-1 genome, and the different sequences were re-amplified to correct the corresponding pepper *Hsf* genes sequences. One candidate gene (Gene ID: CA01g30350) was discarded due to an incomplete DBD domain as identified by Pfam, SMART (http://smart.embl-heidelberg.de/) and Heatster (http://www.cibiv.at/services/hsf/). As a result, 25 *Hsf* candidate genes, whose classification and naming were based on the rules of *Hsf* families from *Arabidopsis* and tomato [1], were identified in pepper (Table 1). The coding sequence sizes for *CaHsfs* ranged from 606 bp (*CaHsfB5*) to 1,518 bp (*CaHsfA1b*), deduced proteins from 201 to 505 amino acids in length, respectively, and molecular weights from 23.37 kDa to 56.06 kDa, respectively. The predicted isoelectric points of CaHsfs were divergent, ranging from 4.65 to 9.20. Among the 25 pepper *Hsf* genes (*CaHsfs*), 17 members belonged to class A (*CaHsfAs*) and seven members belonged to class B (*CaHsfBs*), while only one *Hsf* gene was a class C member (*CaHsfC*). In *CaHsfA*, the subclasses of *CaHsfA9* (four members, *A9a*, *b*, *c* and *d*), *CaHsfA1* (three members, *A1b*, *d* and *e*), *CaHsfA4* (three members, *A4a*, *b* and *c*) and *CaHsfA6* (three members, *A6a*, *b* and *c*) were larger than the other subclasses, while subclass *CaHsfA7* had no members.

**Table 1:** The list of CaHsf members identified

Number	Gene name	Sequenced ID	Chr.	ORF Length (bp)	No. of AA	Mol. Wt. (kDa)	pI
1	CaHsfA1b	CA03g21660	3	1518	505	56.06	5.08
2	CaHsfA1d	CA01g07540	1	1509	502	55.62	4.65
3	CaHsfA1e	Capana06g000777★	6	1443	480	54.18	5.98
4	CaHsfA2	CA08g05000	8	1089	362	41.19	4.69
5	CaHsfA3	CA09g01450	9	1350	449	50.28	4.77
6	CaHsfA4a	CA04g01070	4	1209	402	45.81	5.18
7	CaHsfA4b	CA07g15920	7	1299	432	49.29	5.38
8	CaHsfA4c	CA02g13280	2	1215	404	45.74	5.83
9	CaHsfA5	CA12g20590	12	1362	453	50.92	5.60
10	CaHsfA6a	CA03g06850	3	1089	362	42.30	5.28
11	CaHsfA6b	CA06g08710	6	1011	336	38.75	4.74
12	CaHsfA6c	CA03g11650	3	1080	359	41.13	5.23
13	CaHsfA8	CA09g11190	9	1206	401	46.11	4.75
14	CaHsfA9a	Capana07g000898★	7	1107	368	42.59	5.52
15	CaHsfA9b	Capana12g002488★	12	777	258	30.09	9.20
16	CaHsfA9c	CA02g13320	2	987	328	37.05	6.86
17	CaHsfA9d	CA02g16840	2	1143	380	43.53	4.95
18	CaHsfB1	CA02g11030	2	915	304	33.69	4.99
19	CaHsfB2a	CA03g16300	3	981	326	36.37	5.01
20	CaHsfB2b	Capana01g000273★	1	1008	335	36.37	5.01
21	CaHsfB3a	CA05g00840	5	705	234	27.42	9.13
22	CaHsfB3b	CA10g20440	10	735	244	28.28	8.09
23	CaHsfB4	CA04g18550	4	1140	379	42.92	7.62
24	CaHsfB5	CA02g16000	2	606	201	23.37	9.02
25	CaHsfC1	Capana09g000311★	9	978	325	36.12	6.52

ORF: open reading frame; AA: amino acid; Mol. Wt.: molecular weight; pI: isoelectric point. Pentagram (★) marks that sequenced IDs are from Zunla-1 genome, and others without pentagram from CM334 genome

### Identification of conserved domains in pepper Hsf proteins

**Fig. 1:**
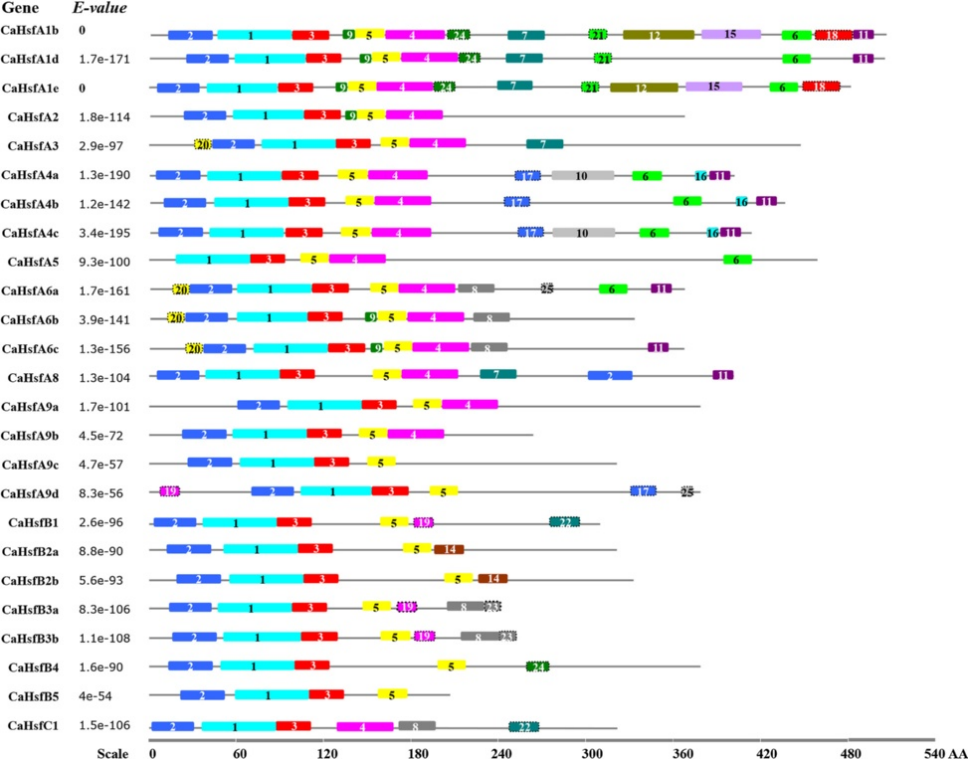
Motifs identified by MEME tools in pepper Hsfs. In total, 25 motifs were identified and are indicated by increasing numbers from 1 to 25. Because motif 13 was the same as motif 8, it was labelled as motif 8. Different motifs are indicated by different borders and colours. The names of the Hsf members from pepper and their combined *P*-values are on the left side of the figure, and the motif sizes are indicated at the bottom of the figure. The same number in different Hsfs refers to the same motif

The MEME web server (http://meme-suite.org/tools/meme) was used to analyze motifs in CaHsf proteins (Fig. 1, Table 2). Motif 1 and 3 were found in all 25 pepper Hsf members, while motif 2 was absent in CaHsfA5 and motif 5 was absent in CaHsfC1. Some motifs only existed in certain members, such as motif 4, which was found in most CaHsfA and CaHsfC members, but not in CaHsfB. Generally, the number of motifs in the CaHsfBs was less than those in the CaHsfAs.

**Table 2:** Motif sequences indentified by MEME tools

Motif	Width	Multilevel consensus sequence
1	50	FIVWDPPEFARDLLPKYFKHNNFSSFVRQLNTYGFRKVDPDRWEFANEWF
2	29	CHGNAPPPFLTKTYEMVDDPSTDDIISWN
3	26	LRGQKHLLCNIHRRKPWHNHSHQCCH
4	41	RQHQQGTDHQIQAMTERLQATEHRQQQMMSFLAKAMQNPGF
5	21	YEEEIERLKREKNVLMMELVK
6	20	VPTGVNDVFWEQFLTERPGC
7	24	DGQIVRYQPPMNEAAKALLQQICK
8	26	QIMQQKGKRKELEEAIRKKRRRPIDH
9	8	CVEVGKFG
10	42	LDESRSCADSPAMSYPQLDIDVGPKVSGIDMNSEPNGNPTPD
11	14	HMNNLAEQMGHLTS
12	48	HLMSDSGFPFNSCLSVMPEIQYSPTVVPGEAKVPQFPELDALNSQIDH
13	6	KKRRLP
14	19	LCGNIYNMMSNYNADCAEI
15	41	YQGHGIDRLDTLGRADFKRSETGNMPDIDTMQGIEDGVTTV
16	6	HKYWWN
17	17	LESSLTFWEHYLYDIDQ
18	29	DEIGSLAVEDGLDKEEDFPGVQESDWDKL
19	13	KCKELIDIVSMFA
20	11	PTMWIPQPMEG
21	12	GGTSPHISGVTL
22	20	CGGRGKMMKANDYYGPWMKM
23	11	IANIFISQLCK
24	15	AQLVHQQNESNRHIT
25	6	PHQYYY

Motif numbers corresponded to the motifs in Fig. 1

To better understand the structural characteristics of the CaHsf family, the conserved domains were predicted using Heatster (Table 3). Six conserved domains, DBD, HR-A/B, NLS, AHA, RD and NES, were identified in three CaHsf classes. As the most conserved domain in the Hsfs, DBD (corresponding to motif 1 and parts of motifs 2 and 3 in Fig. 1) was found in all 25 CaHsf members (Additional file 1: Fig. S1). The DBD domain was composed of three helical bundles (*α*1, *α*2 and *α*3) and four antiparallel *β*-sheets (*β*1, *β*2, *β*3 and *β*4), while no *α*1 and intact *β*1 were detected in the DBD domain of CaHsfA5, which resulted in its sequence being shorter than those of the other CaHsfs. In addition to DBD, HR-A/B, another core conserved domain, was also presented in all CaHsf proteins, while the other four conserved domains were only found in specific CaHsfs members. For the CaHsfAs, the NLS domain was found in all 17 members. CaHsfA9a had the longest NLS sequence (from the 57th to 248th amino acid), which covered the DBD and HR-A/B domains, while CaHsfA9b, A9c and A9d had the shortest NLSs of two amino acids. Three and four AHA domains, the specific domain that characterizes CaHsfAs, were identified in CaHsfA2 and CaHsfA3, respectively, while none were found in CaHsfA9b. For the CaHsfBs, CaHsfB1 and B5 did not contain the NLS domain, and similar to CaHsfA9a, CaHsfB3a also possessed a long NLS sequence (from the 10th to 218th amino acid) covering the DBD and HR-A/B domains. The tetrapeptide motif LFGV, as the core of the RD, was identified in all CaHsfB members except CaHsfB5, but only CaHsfB4 contained the NES domain. Interestingly, only two domains, DBD and HR-A/B, were identified in CaHsfC1.

**Table 3:** Functional domains of CaHsf members in pepper

Gene	Domains					
	DBD(▲)	HR-A/B(◆)	NLS(●)	AHA(★)	RD(■)	NES(✩)
**CaHsfA1b**	▲18-111	◆134-198	●(216) RRITGLNKKRR	★AHA2(441) DIFWDHILSA	nd	✩(492) LTEQMGLL
**CaHsfA1d**	▲31-124	◆146-210	●(222) KHMIERSKKRR	★AHA2(435) DPFWEKFLQN	nd	✩(482) LTEQMEQL
**CaHsfA1e**	▲11-104	◆127-191	●(209) RFITGMNKKRR	★AHA2(428) DIFWDQILLA	nd	nd
**CaHsfA2**	▲29-122	◆137-201	●(217)RKDKQRIEVGQKRR	★AHA1 (274) MLFSAALEN; AHA1(306) ENIWEELL; AHA2(346) PVWGEELED	nd	✩(355)LVDQLGF
**CaHsfA3**	▲49-142	◆167-213	●(239) RTMRKFIKHQ	★AHA1(368) EEEVWSM; AHA2(387) TELWGG; AHA3(406) LSDLWDLDPL; AHA4(423) VDKWPDD	nd	nd
**CaHsfA4a**	▲11-104	◆129-186	●(204) RKRR	★AHA1(256) LTNWEHILYD; AHA2(340) DVFWEQFLTE	nd	✩(389) LTEQLEHL
**CaHsfA4b**	▲17-110	◆133-190	●(208) KKRR	★AHA1(246) INFWEHFLYG; AHA2(367) DVFWEQFLTE	nd	✩(417) LAERMGHL
**CaHsfA4c**	▲11-104	◆131-188	●(206) RKRR	★AHA1(258) LTFWEDVLHN; AHA2(344) DVFWEQFLTE	nd	✩(391) LAEQLGHL
**CaHsfA5**	▲13-84	◆104-161	●(172) QKLESMDISAFSKKRR	★AHA(401) DVFWEQFLTE	nd	✩(441) VASNTRKV
**CaHsfA6a**	▲34-127	◆145-209	●(224) RRKELEEEIRNKKRR	★AHA(318) EGFWERLLSE	nd	✩(347) VDILAHHLGFL
**CaHsfA6b**	▲30-123	◆149-214	●(229) KRKELEEAIKTKRRR	★AHA(300) EGLWEDMLNE	nd	nd
**CaHsfA6c**	▲44-137	◆154-218	●(233) KRKEIEEAITKKRQR	★AHA(322) AGFWEELFNE	nd	nd
**CaHsfA8**	▲12-119	◆147-204	●(326) ITENKEDEV	★AHA1(312) DDDDMLEQLL	nd	nd
**CaHsfA9a**	▲66-158	◆181-231	●(57–248)	★AHA(324) YILWEKLMED	nd	✩(350) IVHELEDLI
**CaHsfA9b**	▲30-122	◆141-191	●(207) MK	nd	nd	nd
**CaHsfA9c**	▲34-126	◆151-201	●(217) TR	★AHA(261) REFWVKLFED	nd	✩(301) IAMEGEALI
**CaHsfA9d**	▲77-169	◆193-243	●(259) VE	nd	nd	nd
**CaHsfB1**	▲7-100	◆155-192	nd	nd	■(251) KLFGVLL	nd
**CaHsfB2a**	▲23-116	◆173-209	●(270) KRIRE	nd	■(261) RLFGFSI	nd
**CaHsfB2b**	▲27-120	◆202-238	●(290) KRVRR	nd	■(281) RLFGVSL	nd
**CaHsfB3a**	▲19-112	◆145-180	●(10–218)	nd	■(200) MLFGVRL	nd
**CaHsfB3b**	▲22-115	◆153-188	●(227) KR	nd	■(210) KLFGVRL	nd
**CaHsfB4**	▲22-115	◆198-234	●(336) KRVH	nd	■(326) KLFGVPL	✩(360) LMVLEKDDLGLNLM
**CaHsfB5**	▲28-125	◆158-198	nd	nd	nd	nd
**CaHsfC1**	▲7-101	◆121-164	nd	nd	nd	nd

DBD (▲): DNA-binding domain; HR-A/B (◆): OD (oligomerisation domain), Heptad pattern of hydrophobic amino acid residues; NLS (●): Nuclear localisation signal; AHA (★): Activator motifs, **a̲**romatic (W, F, Y), large **h̲**ydrophobic (L, I, V) and **a̲**cidic (E, D) amino acid residues; RD (■): Tetrapeptid motif LFGV as core of repressor domain; NES (✩): Nuclear export signal. Numbers in brackets indicates the position of the first amino acid present in the putative NLS, AHA, RD and NES in the C-terminal. nd: no domains detectable by sequence similarity searches by Heatster

### Phylogenetic and sequence structure analysis in pepper Hsf proteins

**Fig. 2:**
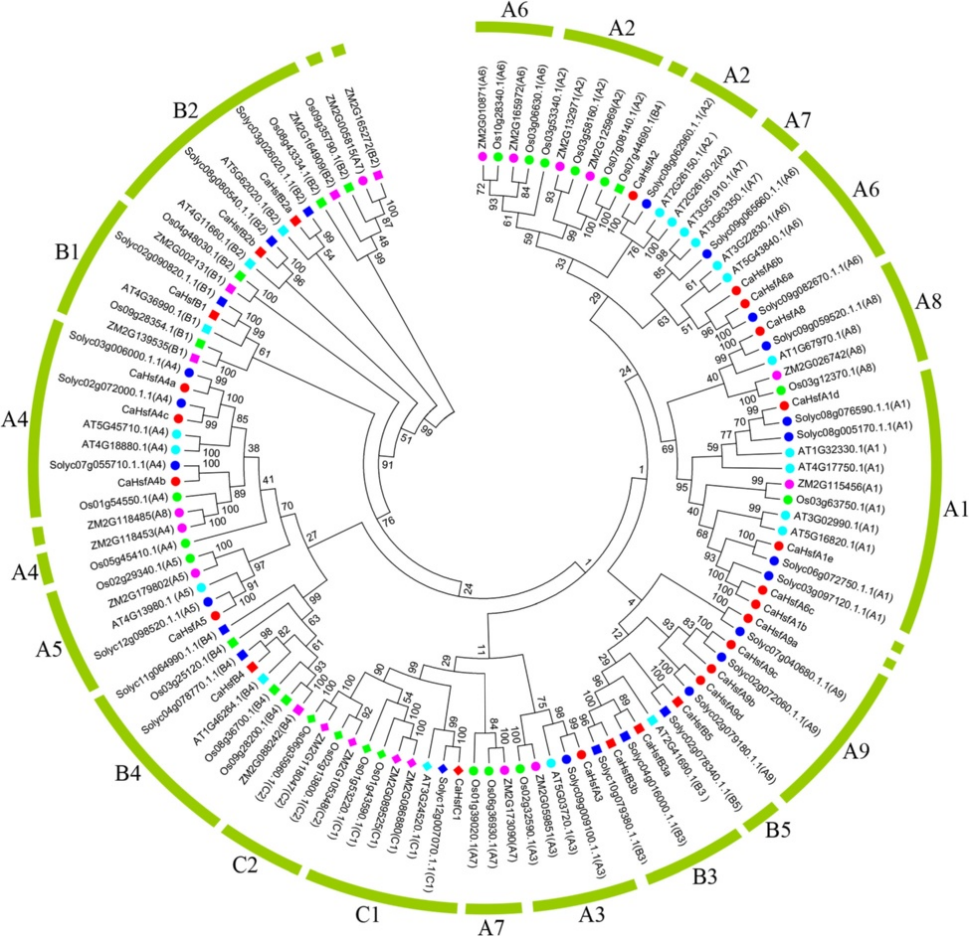
Neighbour-Joining phylogenetic tree of Hsf proteins from pepper, tomato, *Arabidopsis*, rice and maize. The N-proximal regions (from the start of the DNA-binding domain to the end of the HR-A/B region) of Hsf proteins were used to construct of the phylogenetic tree with MEGA 5.10. For *Arabidopsis* (prefixed by AT), tomato (prefixed by Solyc), rice (prefixed by Os) and maize (prefixed by ZM) Hsf proteins, both locus ID and subclass numbers are listed. CaHsf proteins are marked in red. An unrooted Neighbour-Joining analysis was performed with pairwise deletion and Poisson correction

To discover the phylogenetic relationships among the Hsf families, the Hsf conserved amino acid sequences (from the start of the DBD domain to the end of the HR-A/B region) [1, 41] of 25 proteins from pepper, 21 from *Arabidopsis*, 25 from tomato (*S. lycopersicum*), 21 from maize (*Z. mais*) and 25 from rice (*O. sativa*) were used to generate a phylogenetic tree (Fig. 2). Based on the phylogenetic tree, class HsfA had the maximum number of subclasses among the three classes, and included five smaller clusters of which four (A2 and A6, A1 and A8, A9, A3 and A7) were closer to class HsfC than the fifth cluster of class HsfA (A4 and A5). Two HsfA7 members from *Arabidopsis* (AT3G51910.1 and AT3G63350.1) were not clustered with the HsfA7 subclass from other plant species, but were closer to the HsfA6 subclass. This was also observed for one member from the maize HsfA7 subclass (ZM2G005815, closer to subclass HsfB2), one member from the maize HsfA8 subclass (ZM2G118485, closer to subclass HsfA4), one member from the rice HsfB4 subclass (Os07g44690.1, closer to subclass HsfA2) and CaHsfA6c (closer to subclass HsfA1). Compared with *Arabidopsis*, maize and rice, tomato Hsfs were closer to pepper Hsf proteins, which was coincident with the botanical classification.

A phylogenetic tree based on the sequences of conserved domains (from DBD to HR-A/B) in pepper Hsfs was also constructed (Additional file 2: Fig. S2A), which corresponded to the above mentioned motifs distributions (Fig. 1, Table 2) and phylogenetic groups (Fig. 2). The exon/intron structure of all 25 pepper Hsf members was analysed based on their coding sequences and the corresponding genome sequences to obtain further insights into duplication events and evolutionary patterns. *CaHsfs* shared a highly conserved exon/intron structures, with one intron and zero intron phases (Additional file 2: Fig. S2B). There were 15 CaHsf members with the intron located in the DBD domain, and four members with the intron located between the NLS and AHA domains, while the introns in CaHsfA4a and A4b were located between the AHA1 and AHA2 domains. The length of introns varied from 77 bp (*CaHsfB2a*) to 3,205 bp (*CaHsfA5*).

### Chromosomal location and Hsf gene duplications in the pepper genome

To determine the chromosomal distribution of the *CaHsf* genes, the positions were identified based on their physical positions in the pepper genome database PGP. The 25 members mapped to 11 out of the 12 pepper chromosomes, with no genes mapping to chromosome 11 (Additional file 3: Fig. S3). The number of *CaHsf* genes on each chromosome varied greatly. The largest number of *CaHsf* genes (5) was located on chromosome 2, four genes were identified on chromosome 3, three genes on chromosome 9, and two genes each on chromosomes 1, 4, 6, 7 and 12. There was only one *CaHsf* gene each on chromosomes 5, 8 and 10.

The Plant Genome Duplication Database (PGDD, http://chibba.agtec.uga.edu/duplication/) analysis confirmed that two pairs of the pepper *Hsfs* (*CaHsfA4a*/*A4c* and *CaHsfB3a*/*B3b*) were segmental duplicated sequences (Additional file 3: Fig. S3, Additional file 4: Table S1), and each of the two pairs were located on different chromosomes (the former on chromosomes 2 and 4, and the latter on chromosomes 5 and 10). The ratios of nonsynonymous to synonymous substitutions (Ka/Ks) for the two duplicated pairs were less than 1.0, which suggested that the pairs had evolved mainly under the influence of purifying selection and that the duplication events occurred 45.9 (*CaHsfA4a*/*A4c*) and 71.31 million years ago (*CaHsfB3a*/*B3b*) [44, 45].

### Protein–protein interaction network among CaHsf members

To provide further biological information on CaHsf members, their protein–protein interaction network of CaHsfs was predicated based on the interolog from the *Arabidopsis* interactome. Every CaHsf member, except CaHsfA5, generated a complex interaction network (Additional file 5: Fig. S4). The *Arabidopsis* homolog of CaHsfA5 (AtHsfA5, At4g13980) was not found among the *Arabidopsis* Hsf interaction partners. Among the CaHsfA members, A2, A3 and A6 (A6a, A6b and A6c) interacted with most other CaHsfs, and the three CaHsfA1 members (CaHsfA1b, A1d and A1e) also interacted with each other. However, CaHsfB1 (9 interaction partners), B3a (6 interaction partners) and B3b (6 interaction partners) owned the simple interaction network compared with class CaHsfA and other class CaHsfB members, and they did not interact with CaHsfA2, A1 and A6 members. In general, the class CaHsfA members had more interaction partners than class CaHsfB and CaHsfC members.

### Expression analysis of *CaHsf* genes at different developmental stages in various organs

**Fig. 3:**
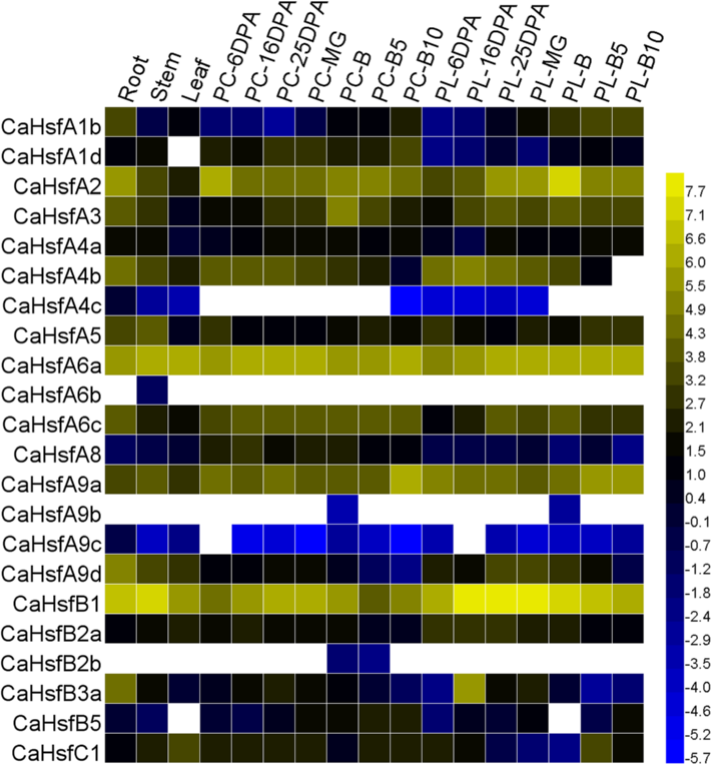
Tissue-specific expression analysis of pepper *Hsf* genes. Raw data were from RNA-seq data of each tissue from CM334. The analysed tissues including root, stem, leaf, pericarp (PC) and placenta (PL) at 6, 16, 25 days post-anthesis (DPA), PC and PL at mature green (MG) and at breaker (B) stages, PC and PL at 5 and 10 days post-breaker (B5 and B10, respectively). The data from 22 pepper *Hsf* genes (excluding *CaHsfA1e*, *B3b* and *B4* whose date are absent) were used to create a heat map using HemI. The RNA-seq data for zero is indicated as white, and the other data were normalized using log2

To investigate the potential functions of *CaHsf* genes during pepper development, a heat map of the global transcription patterns of the *CaHsf* family in CM334 was generated for the pepper genes against RNA-seq data of five tissues (root, stem, leaf, pericarp and placenta) and seven developmental stages of pericarp and placenta (Fig. 3). The expression pattern of each *CaHsf* gene was significantly different in different tissues and stages. Among class *CaHsfA*, *CaHsfA2*, *A6a* and *A9a* were constitutively expressed at relatively high levels, while *CaHsfA4c*, *A6b*, *A9b* and *A9c* were expressed at low levels or undetectable in all tested tissues, and the remained class *CaHsfA* genes were expressed highly in some specific tissues. For example, the expression levels of *CaHsfA4b* in root, PL-6DPA (placenta at 6 days post-anthesis) and -16DPA were higher than those in other tissues, but undetectable in PL-B10 (placenta at 10 days post-breaker). Although constitutively expressed, *CaHsfA2*, *A6a* and *A9a* were transcribed with higher levels in reproductive organs [PL-B (placenta at breaker) for *CaHsfA2*, PC- and PL-MG (pericarp and placenta at mature green) for *A6a*, and PC-B10 (pericarp 10 days post-breaker) for A9a].

*CaHsfB1* was also constitutively expressed in all tested tissues at relatively high abundances, especially in PL (including PL-16DPA, −25DPA, −MG and -B). In comparison, the expression level of *CaHsfB2b* was at its lowest levels in PC-B (pericarp at breaker) and -B5, and undetectable in other tissues. *CaHsfB3a* was expressed at a higher level in PL-16DPA than in other organs and other PL developmental stages. In vegetative and reproductive organs, the highest levels of *CaHsfC1* were observed in leaf and PL-B5, respectively, and the lowest level was found in PL-B.

### Expression analysis of *CaHsf* genes under HS treatment

**Fig. 4:**
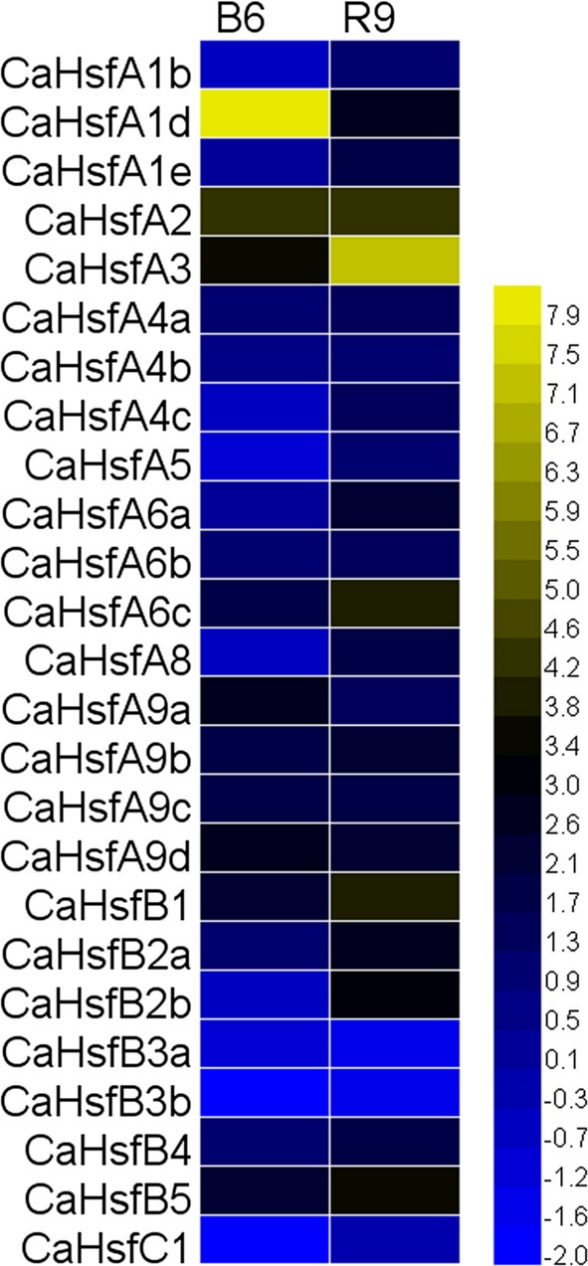
Relative gene expression levels of *CaHsfs*, analysed by qRT-PCR, in response to HS treatment in B6 and R9 leaves. HS treatment: 40 °C for 2 h; B6: pepper thermosensitive line; R9: pepper thermotolerant line. qRT-PCR data were normalized using the pepper ubiquitin-conjugating protein gene (*UBI-3*) and are shown relative to 0 h. The relative expression levels were calculated using the -ΔΔCT method and then a heat map with HemI was created

To examine the heat response profile for *CaHsfs* in pepper, we analysed the transcription levels of *CaHsf* members in the leaves of thermosensitive line B6 and thermotolerant line R9 under HS condition (40 °C for 2 h) [37, 38]. As shown in Fig. 4, in the heat-stressed R9 leaves, 22 *CaHsf* genes (88 %) were up-regulated (>2-fold) by HS, and two members, *CaHsfB3a* and *B3b*, were down-regulated (<0.5-fold), while only *CaHsfC1* did not show a marked change. Among the up-regulated members, the expression levels of *CaHsfA2*, *A3*, *A6c*, *B1* and *B5* were higher than other members under HS, and the greatest increase in expression (>140-fold) was found in *CaHsfA3*, followed by *CaHsfA2* (~20-fold). Compared with other groups, *A1* members (*CaHsfA1b*, *A1d* and *A1e*) were not the predominantly expressed *CaHsf* genes. The transcription levels of the two pairs of duplicated *CaHsf* genes (*CaHsfA4a*/*A4c* and *CaHsfB3a*/*B3b*) did not exhibit significant divergences in regulated expression under HS. For the thermosensitive line B6, only 13 genes (52 %) were up-regulated and 10 genes (40 %) maintained a stable expression level under HS conditions, which was more than in R9. *CaHsfA1d*, *A2* and *A3* were strongly induced in treated B6 leaves, and *CaHsfA2* and *A3* showed particularly strong responses to HS in both thermosensitive line B6 and thermotolerant line R9 (Fig. 4).

### Expression profiles of *CaHsf* genes in response to salt and osmotic stress

Although it was well known that Hsfs are involved in plant heat acclimatisation, other adverse factors, like salt and osmotic stresses, also affected plant growth and development, so we wondered whether responses to these stresses involved *CaHsfs*. Transcription profiles were obtained for *CaHsf* genes in R9 roots and stems subjected to 300 mM NaCl (salt stress) for 6 h, and 5 % mannitol (osmotic stress) for 6 h, respectively.

**Fig. 5:**
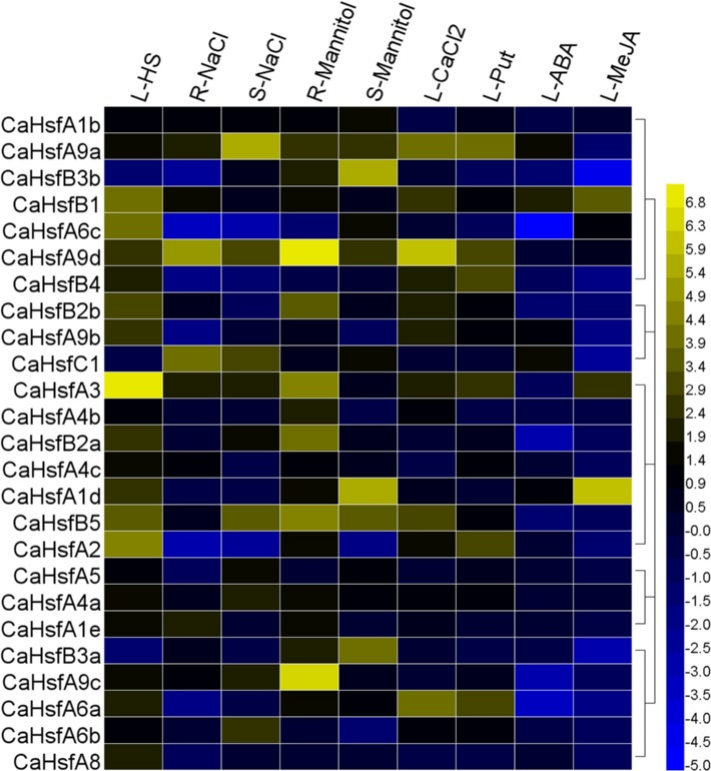
qRT-PCR analysis of relative *CaHsfs* transcript levels in R9 plants exposed to various abiotic stresses. qRT-PCR data of HS as seen in Fig. 4. R9: pepper thermotolerant line; L-HS: heat stress (40 °C for 2 h); R-NaCl and S-NaCl: salt stress (300 mM NaCl for 6 h) responsiveness of *CaHsf* genes in roots and stems, respectively; R-Mannitol and S-Mannitol: osmotic stress (5 % mannitol for 6 h) responsiveness of *CaHsf* genes in roots and stems, respectively. The expression levels under salt and osmotic stress treatments were relative to that of the samples treated with water. L-CaCl_2_, −Put, −ABA and -MeJA: CaCl_2_ (15 mM for 6 h), putrescine (Put, 1.5 mM for 6 h), abscisic acid (ABA, 100 μM for 3 h) and methyl jasmonate (MeJA, 100 μM for 6 h) responsiveness of *CaHsf* genes in leaves, respectively. MeJA was dissolved in 10 % ethanol and other substances were dissolved in water; therefore, control seedlings were sprayed with 10 % ethanol (for the MeJA treatment) or water (for the CaCl_2_, Put and ABA treatments). The expression levels are relative to that of the control samples

Under the salt stress treatment, six members (*CaHsfA1b*, *A3*, *A9a*, *A9c*, *A9d* and *C1*) were up-regulated in roots and stems, while *CaHsfA2*, *A6c* and *B4* were down-regulated in both tissues (Fig. 5). The expression levels of five genes, *CaHsfA1d*, *A4b*, *A8*, *B2b* and *B3a*, were unregulated in roots and stems, while the expression levels of the remaining 11 members only showed obvious changes in either roots or stems. For instance, *CaHsfA1e*, *A4c* and *B1* were induced by salt stress in roots, but not in stems; however, *CaHsfA4a*, *A5*, *A6b* and *B5* exhibited high expression levels only in stems. Interestingly, in the subclass of *CaHsfA9, CaHsfA9d* was strongly induced (>30-fold) by salt stress in roots, while *CaHsfA9a* from stems seemed to be more sensitive to salt stress (~40-fold).

Under osmotic stress, nine members (*CaHsfA1b*, *A1d*, *A4a*, *A6a*, *A9a*, *A9d*, *B3a*, *B3b* and *B5*) were up-regulated, while four members (*CaHsfA4c*, *A8*, *A9b* and *B4*) were unregulated in stems and roots, and the highest expressing *CaHsf* genes were *CaHsfA9d* (>160-fold) in root and *CaHsfB3b* (~46-fold) in stem. However, no gene was down-regulated in both roots and stems. It is noteworthy that the expression of *CaHsfA1b*, *A9a* and especially *A9d* could be induced by both salt and osmotic stresses in both stems and roots.

### Expression profiles of *CaHsf* genes responses to exogenous ABA, MeJA, putrescine (Put) and CaCl_2_

Phytohormones and plant signalling molecules, such as ABA, MeJA, Put and Ca^2+^, are involved in various stress signalling pathways [6, 37, 46]. To explore the responses of *CaHsf* family genes to these signals, we analysed the expression profiles of *CaHsf* genes in R9 leaves treated with these exogenous substances. As shown in Fig. 5, after a CaCl_2_ treatment, 13 *CaHsf* genes were significantly up-regulated, while 12 genes were unregulated. Similarly, 11 *CaHsf* genes were unregulated by a Put treatment, and no gene was down-regulated. Only five and four of the 25 *CaHsf* genes were up-regulated by ABA and MeJA treatment, respectively, whereas seven and nine members were down-regulated, respectively.

*CaHsfB1* expression could be induced by all four signal substances, while *CaHsfA9a* and *A9b* were up-regulated by CaCl_2_, Put and ABA, but down-regulated by the MeJA treatment. In addition, *CaHsfA6a* was induced by CaCl_2_ and Put, but down-regulated by ABA and MeJA. The genes with the highest induced levels by CaCl_2_, Put and MeJA treatment were *CaHsfA9d*, *CaHsfA9a* and *CaHsfA1d*, respectively; however, the transcriptional levels of *CaHsfs* after the ABA treatment were not as high as in other three treatments. The highest expression level of *CaHsfB1* induced by ABA increased less than 5-fold compared to the control.

### CaHsfA2 locates to the cellular nucleus

**Fig. 6:**
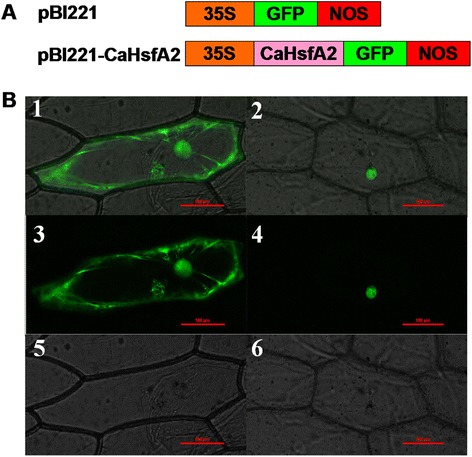
Transient expression of the CaHsfA2-GFP fusion protein in onion epidermal cells. (**a**) Schematic diagram of the construction of the recombinant CaHsfA2-GFP vector. 35S: a constitutive promoter from the cauliflower mosaic virus; GFP: green fluorescent protein; NOS: nopaline synthase terminator. (**b**) Transient expression of GFP and CaHsfA2-GFP in onion epidermal cells. (**b** ***1***, **b** ***3***, **b** ***5***) Onion epidermal cells transformed with 35S::GFP as control. (**b** ***2***, **b** ***4***, **b** ***6***) Onion epidermal cells transiently expressing 35S::CaHsfA2-GFP. (**b** ***1***, **b** ***2***) Merged images. (**b** ***3***, **b** ***4***) Dark field images. (**b** ***5***, **b** ***6***) Bright field images. Bars = 0.1 mm

Because of its dominant role in thermotolerant cells [1] and significantly up-regulated expression (Fig. 4), we characterized CaHsfA2 in pepper. First, to clarify whether the CaHsfA2 protein localizes to the nucleus, we investigated the cellular localization of CaHsfA2 protein in a transient expression assay by introducing the 35S::CaHsfA2-GFP (pBI221-CaHsfA2-GFP) translational fusion into onion epidermal cells using particle bombardment. The fluorescence of cells transformed with the control 35S::GFP (pBI221-GFP) was distributed throughout the cell, including the nucleus, cytoplasm and cytomembrane. In contrast, the fluorescence of the 35S::CaHsfA2-GFP chimera was associated with the cellular nucleus in onion epidermal cells, suggesting a nuclear localization of CaHsfA2 (Fig. 6).

### CaHsfA2 shows transcriptional activity

**Fig. 7:**
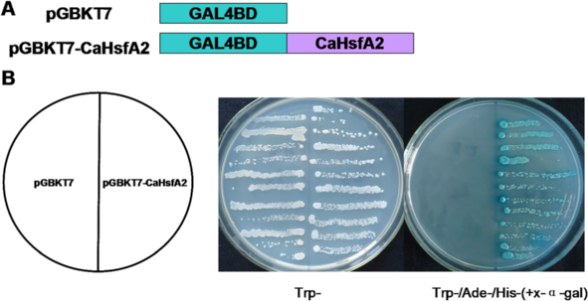
Transactivational activity of the CaHsfA2 protein in yeast. (**a**) Schematic diagram illustrating the *CaHsfA2* cDNA fragments encoding CaHsfA2 that was fused to the DNA sequences encoding the GAL4 DNA binding domain in the yeast vector pGBKT7. (**b**) Transactivational analysis of CaHsfA2 in yeast. Fusion proteins of pGBKT7-CaHsfA2 and pGBKT7 were expressed in yeast strain AH109. The transformants were streaked on the SD/Trp- and SD/Trp-Ade-His- (with X-α-gal) medium. The plates were incubated at 30 °C for 3 d

The transcriptional activity of the CaHsfA2 protein was examined using a yeast expression system. The fusion plasmids pGBKT7-CaHsfA2 and pGBKT7 (control) were transformed into yeast strain AH109, and grown on SD medium lacking tryptophan (SD/Trp-) or lacking tryptophan, histidine and adenine (SD/Trp-Ade-His-). The growth status of transformants was evaluated (Fig. 7). Yeast cells containing either pGBKT7 or pGBKT7-CaHsfA2 could grow well on SD/Trp- plates; however, only cells containing pGBKT7-CaHsfA2 could grow on SD/Trp-Ade-His- plates and turn blue in the presence of 5-bromo-4-chloro-3-indoxyl-α-D-galacto-pyranoside (X-α-gal), which showed that *LacZ*, the second reporter gene, was activated by CaHsfA2. The above results demonstrated the presence of transcriptional activity in the CaHsfA2 protein.

### *CaHsfA2* discriminatively responds to HS in two pepper lines differing in thermotolerance

**Fig. 8:**
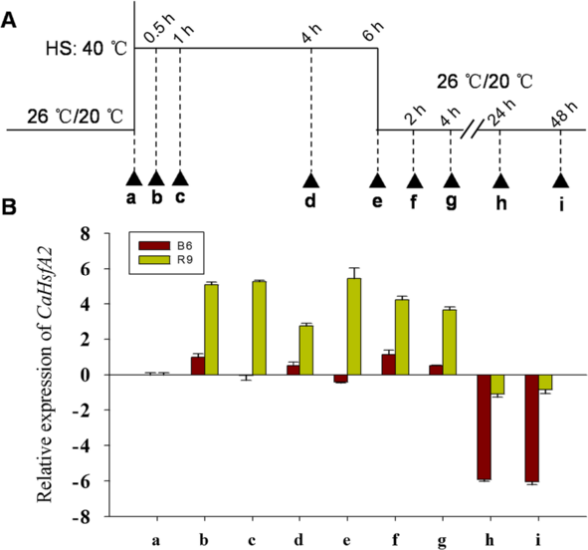
Expression level of *CaHsfA2* in pepper in responses to HS treatment. B6: pepper thermosensitive line; R9: pepper thermotolerant line. (**a**) The time course of HS treatments. Triangles indicate the time points when the leaves were collected (sample *a*-*i*). (**b**) Expression profiles of *CaHsfA2* in pepper leaves under the HS treatment at different time points. Expression data were normalized with *UBI-3* as the reference gene. The expression levels are relative to that of the sample *a* from B6 and R9, respectively

To further determine the response of *CaHsfA2* to HS, we analysed the expression of this gene under HS and during recovery at room temperature in thermosensitive line B6 and thermotolerant line R9 (Fig. 8a). During HS, although the *CaHsfA2* level was induced after 0.5 h at 40 °C (Fig. 8b, sample *b*) in B6 and R9, the expression level in R9 leaves was maintained at a higher level (~40-fold) compared with control to the end of the HS treatment (40 °C for 6 h) (Fig. 8b, sample *a*). After the heat-treated seedlings were moved back to normal temperature conditions for the 48 h recovery treatment, the *CaHsfA2* expression level remained at high after a short recovery time (from 2 to 4 h) in R9, while it was down-regulated after a long recovery time (from 24 to 48 h) in both B6 and R9 leaves (Fig. 8b, samples *h* and *i*). It is worth noting that the *CaHsfA2* expression level in R9 leaves under HS conditions for 4 h (sample *d*) was lower than after a 1 h HS treatment(sample *c*), and the level was slightly down-regulated at the end of the HS treatment (sample *e*) in B6.

## Discussion

More and more evidence suggests that Hsfs play central roles in plant developmental and defence processes [5, 12, 14, 31, 32, 36, 47]. Benefiting from genome availability, the functions of the *Hsf* family genes have been characterized in many plants, including the model plants *Arabidopsis* [20, 48], maize [8] and rice [39], as well as other plants, including grass [49], Chinese cabbage [40] and apple [50]. However, with the limited investigations into the molecular basis of heat tolerance, little is known about the *Hsf* family in pepper.

In the present study, 25 *Hsf* genes in the nutritionally and economically important pepper were identified based on the pepper genome (Table 1) [42, 43]. Although the total number of *Hsf* genes was similar to that of *Arabidopsis* and tomato [1], the members of some specific *Hsf* subclasses in pepper were different from other two species. For example, the number of subclass *HsfA1* members in pepper was less, but the number in subclass *HsfA6* was more, than in tomato, while no pepper *Hsf* members were classified into subclass *HsfA7*, which suggested the possibility of a gene loss event during the evolutionary process [39]. Another interesting observation was the surprising enlargement of the subclass *CaHsfA9* with four members in pepper, compared with only one member in tomato. Usually a single *HsfA9* gene is found in eudicots, including *Arabidopsis* and tomato, whereas soybean contained two members and *Eucalyptos grandis* (*Myrtaceae*) contained at least 17 closely related *HsfA9*-encoding genes [1]. The reasons for the expansion of the *CaHsfA9* genes remain to be elucidated by further investigations.

The DBD domain of about 100 amino acid residues is highly conserved in yeast, mammals and plants [18]; however, it is noteworthy that the DBD of CaHsfA5 in pepper, having only 72 amino acid residues, was shorter than the other CaHsfs, lacked the full *α*1-helix and had a truncated *β*1-sheet. However, the central helix-turn-helix motif (*α*2-turn-*α*3) required for specific interactions with HSEs, and the W amino acid residue in the incomplete *β*1-sheet required for aromatic-aromatic interactions [51], were still intact in the DBD domain of CaHsfA5, which might allow the protein to exert basic functions, but the effects of the truncated DBD in CaHsfA5 need to be determined. It was interesting that AHA, the essential domain for activator function in the HsfA class [1], did not occur in CaHsfA9b and A9d (Table 3). These Hsfs that lacked AHA domains could play their roles using a characteristic pattern of tryptophan residues by providing additive contributions to the activator function or by binding to other HsfAs to form hetero-oligomers [39, 52].

The phylogenetic analysis revealed that pepper Hsf members were more closely related to those from tomato than to those from *Arabidopsis*, maize and rice (Fig. 2), which was consistent with the fact that both pepper and tomato were members of the Solanaceae family [42]. In the HsfA9 subclass, four CaHsfA9 members (CaHsfA9a, A9b, A9c and A9d) and three tomato members (Sl07g040680, Sl02g072060 and Sl11g008410) clustered in the branch of the HsfA9 group. Only one HsfA9 member from tomato was confirmed, while the other two members, Sl02g072060 and Sl11g008410, were identified to be Hsf-like (*Hsfl*) genes, *Hsfl1* and *Hsfl2*, respectively [1]. No Hsf members from eudicot species (*Arabidopsis*, pepper and tomato) clustered in the HsfC2 branch, because gene duplications in the monocots lineage led to subclass HsfC2 being unique in monocot species [1, 41], which was the most marked difference between monocots and eudicots. Conversely, HsfA9, B3 and B5, which emerged presumably after the split of monocots and eudicots [1], were not found in monocot plants (Fig. 2). The structural analyses showed that all of the *CaHsf* genes contained only one intron (Additional file 2: Fig. S2B), which was presumed to be a conservative evolutionary pattern, while the diverse length of the inserted introns might influence the functionally divergences of the *CaHsf* genes.

The *CaHsf* genes were distributed on 11 out of 12 chromosomes, except chromosome 11. Similarly, chromosomes 1 and 5 in tomato lacked *Hsf* genes, suggesting that the *Hsf* genes might distribute widely in the genome of the common ancestor of these members of the Solanaceae family. Gene duplication is a major evolutionary mechanism in genomes that helped plants adapt to various environmental stresses [44]. Two pairs of paralogs (*CaHsfA4a*/*A4c* and *CaHsfB3a/B3b*) were detected in the pepper *Hsf* family, which was less than the number in rice (9 pairs) [39], maize (9 pairs) [8], and apple (12 pairs) [50]. Neither pair was involved in the regional duplications within the same chromosome. The two members of each pair originated from segmental duplications between chromosomes, which occurred at 45.9 (*CaHsfA4a* and *A4c*) and 71.31 million years ago (*CaHsfB3a* and *B3b*). Because most plants with diploidized polyploids retained numerous duplicated chromosomal blocks within their genomes, segmental duplication occurred more frequently than the other two principal evolutionary patterns (tandem duplication and transposition events) in plants [44, 53]. Based on the vital role of segmental duplications in the family evolution, which occurred frequently in slowly evolving gene families [53], we proposed that the pepper *Hsf* gene family might have a slow evolutionary rate, as in maize [8].

The life activities in plants are attributable to protein–protein interactions. The construction of protein–protein interaction networks would provide necessary information on the mechanisms of life activities and on exploring the biological functions of unknown proteins. Based on the interolog from *Arabidopsis*, the protein–protein interaction network among the CaHsfs was constructed (Additional file 5: Fig. S4). The number of CaHsfA interaction partners was the greatest among the three CaHsf classes, which might indicate that CaHsfAs have a unique role as the master regulators of thermotolerance, and were essential for plants survival under serious HS conditions [1, 26]. In the complex network, CaHsfA1s (A1b, A1d and A1e) interacted with CaHsfA2 and these proteins might act synergistically to form a super-activator complex, which strongly regulates downstream HS-related genes in *Arabidopsis* [31]. Despite not participating in the complicated interaction network, the *Arabidopsis* homolog of CaHsfA5 (AtHsfA5) specifically interacted and suppressed the anti-apoptotic factor AtHsfA4 [54], which acted as a pro-apoptotic factor. CaHsfA5 might have a similar function in pepper, although such a role needs to be confirmed.

Gene expression patterns are usually closely related to their functions [39]. In this study, the expression profiles of each *CaHsf* gene in five different tissues were investigated. *CaHsfA2*, *A6a*, *A9a* and *B1* were found to be constitutively expressed at relatively high levels in the various tissues and at multiple developmental stages under normal conditions (Fig. 3). A similar situation was found in other plants, like *Arabidopsis* (A1-type *Hsfs*) [55], apple (*MdHsfA1a*, *A1d*, *B1a* and *B1b*) [50], wheat (*A1* and *A8* groups, and some *A2* and *A6* group members) [41]. The tissue- and stage-specific expression patterns of pepper *CaHsf* family genes, such as *CaHsfA1b* in root, *CaHsfB3a* in PL-16DPA, *CaHsfB5* in PC-B10, and *CaHsfC1* in leaf and PL-B5, indicated that CaHsfs might be widely involved in the development of various organs and tissues, which is helpful to further understanding the functions of *CaHsf* genes in pepper developmental biology [56].

Most of the *CaHsf* genes were up-regulated under HS conditions. *CaHsfA1d*, *A2*, *A3*, *A6c*, *B1* and *B5* were the main members with significantly higher expression levels during HS in B6 and R9 leaves (Fig. 4), which suggested that these CaHsfs were major transcription factors of heat-induced *Hsp* genes under HS conditions [41]. In tomato, the close relative of pepper, HsfA1a acted as master regulator for triggering the heat response and acquired thermotolerance, and could not be replaced by other Hsfs [26]. Among *CaHsfA* members in thermotolerant line R9, the expression levels of three members of subclass *CaHsfA1* (*A1b*, *A1d* and *A1e*) were not striking, compared with *A2*, *A3* or *A6c*, and, similarly, no master regulator was found among the four members of *HsfA1* from *Arabidopsis* yet [1]. These results indicated that there were species-specific features in the functions of Hsf members in regulating genes involved in plant HS responses. The number of up-regulated *CaHsfA* in R9 (17) under HS conditions was greater than in the thermosensitive line B6 (10), and the expression levels of major transcription factors (*A2*, *A3*, *A6c*, *B1* and *B5*) in R9 were higher than their corresponding levels in B6, although the *CaHsfA1d* expression level in B6 was much higher than in R9. These highly expressed *CaHsfAs* might co-regulate the downstream HS-related genes, thus enhancing the pepper’s thermotolerance.

Notably, five out of seven members of class *CaHsfB,* especially *B1* and *B5,* were significantly up-regulated by HS in R9 leaves, which was in accord with the *HsfB1* and *B2* groups in wheat [41]. Most HsfBs, except HsfB5, contain the tetrapeptide LFGV in the C-terminal domain, which is assumed to function as a repressor motif in the transcriptional machinery [8]. The expression patterns of most *CaHsfB* members under HS could be explained by a report in tomato. This work indicated that HS-induced HsfB1 could act as a coativator, cooperating with HsfA1a by forming a ternary complex with histone acetyl transferase HAC1 to synergistically activate a reporter gene [57] or interacted with HsfA1a or A2 to regulate the different stages of HS response [1]. However, the roles of these up-regulated *CaHsfB* members during HS still needs further investigations. Both *CaHsfB3a* and *B3b* were down-regulated in B6 and R9 leaves under HS conditions, which suggested that the paralogs might act as repressors among *CaHsf* members. The expression level of *CaHsfC1* did not present marked changes under HS in the thermotolerant line R9, which was similar to *ZmHsf-13* from the maize *HsfC* class [8], while the down-regulated expression pattern of *CaHsfC1* in the thermosensitive line B6 under HS was similar to those of C1 group members from wheat [41]. The contradictory observation might be attributed to the species- and lines-specific responses to HS in *HsfC*, but testing this hypothesis requires further research.

In addition to HS, *CaHsf* genes were also regulated by salt and osmotic stresses (Fig. 5). *CaHsfA1b*, *A9a* and *A9d* could be induced by HS, salt or osmotic stresses, while *CaHsfA6c* and *B4* were inhibited by salt stress but enhanced by HS. The expression of *CaHsfB3a* and *B3b* increased under osmotic stress but decreased under HS, which indicated that this pair of paralogs might have a specific regulatory role in strengthening a plant’s adaptability to stresses other than HS [41]. Interestingly, the expression pattern of *CaHsfA2* was different under salt stress, HS and even in roots and stems from R9 under osmotic stress; however, the overexpression of *AtHsfA2* conferred not only thermotolerance, but also salt and osmotic stress tolerance [33], which implied that CaHsfs might play different roles in various tissues under different abiotic stresses and that some CaHsfs might participate in shared roles among various stresses [56].

The signaling substances Ca^2+^, Put, ABA and MeJA are involved in many signal transduction pathways under various stress conditions, and regulated *CaHsfs* expression. Different signals regulated different *CaHsfs*. For instance, both Ca^2+^ and Put could up-regulate *CaHsfA6a*, *A9a* and *A9d*, while ABA and MeJA down-regulated *CaHsf*A6a, which indicated that although most CaHsfs were highly conserved, they might play their roles via different signal transduction pathways. Ca^2+^, as the second messenger, couples extracellular signals with intracellular physiological and biochemical reactions to regulate the process of signal transduction in plant cells under abiotic stress. In *Arabidopsis*, calmodulin AtCaM3 was required in HS signalling to activate calcium/calmodulin-binding protein kinase (CBK), and the latter could phosphorylate HsfA1a [6]. Thus, it was inferred that the induction of *CaHsfA6a*, *A9a* and *A9d* by Ca^2+^ might be attributed to CaM3 in pepper through the calcium-signalling pathway. Put can enhance a plant’s tolerance to abiotic stresses by regulating cell pH, balancing reactive oxygen metabolism and stabilizing membrane structures [58], but there were few reports about the function of Put on the regulation of *Hsfs*. In this study, Put induced the expression of *CaHsfA2*, *A3*, *A6a*, *A9a*, *A9d* and *B4*, which could explain our previous observation that Put was involved in the HS process [37]. ABA [59–61] and MeJA [62, 63] have been reported to participate in the protection against heat damage. *CaHsfA6a* was down-regulated by ABA but up-regulated by Ca^2+^ and Put, which suggested that *CaHsfA6a* played different roles in the physiological processes mediated by ABA and the Ca^2+^ or Put pathways. Although the promoters of *CaHsfA1b*, *A4a* and *A4b* contained the CGTCA- or TGACG-motifs (*cis*-acting elements involved in the MeJA-responsiveness) (data not shown), MeJA could barely influence the expression of these genes, which might result from the joint effects of other *cis*-elements and complex regulatory mechanisms.

HsfA2, as the HS-induced enhancer of thermotolerance [1], has been researched in many species [5, 23, 31]; however, only limited characteristics of *HsfA2* in pepper have previously been described [38]. In this study, we found that CaHsfA2 possessed the typical features of the Hsf family, including being located in the nucleus by its NLS domain (Fig. 6, Table 3), having transcriptional activity (Fig. 7), and responding to continuous HS (Fig. 8), which confirmed our genome-wide identification of the Ca*Hsf* genes. Among the 25 pepper *Hsfs*, the induced expression of *CaHsfA2* in response to HS was only less than *CaHsfA3* in R9 or *CaHsfA1d* in B6 (Fig. 4), which suggested that CaHsfA2 might become a dominant Hsf, as seen with *HsfA2* from *Arabidopsis* and tomato [64]. The lower *CaHsfA2* expression level in the thermosensitive line B6 than in the thermotolerant line R9 (Fig. 8) induced under HS might be the reason for the differing thermotolerance levels in the two pepper lines. However, the *CaHsfA2* level in R9 after 4 h of HS was lower than after 1 h, and the level was slightly down-regulated at the end of HS treatment in B6. The different expression pattern might be attributed to regulatory genes or the circadian clock. Under long-term HS conditions, when *Hsp17-II* were maintained at a high level, they directly interacted with *HsfA2*, forming inactive complexes [1]. Once the molecular chaperones were lacking, *HsfA2* would be released from the complexes to act as a transcription factors. In addition, *HsfB2b* was induced after HS and repressed *HsfA2*. However, *CaHsfA2* might be a rhythmic gene, like *Arabidopsis HsfB2b*, which is regulated by both HS and the circadian clock [65], while the regulatory mechanism of *CaHsfA2* responding to HS needs to be explored further.

## Conclusions

In this study, we identified a total of 25 *CaHsfs* in the pepper genome. Based on the bioinformatics analysis of highly conserved domains, the *CaHsf* genes were divided into three classes, class CaHsfA, B and C, and distributed in 11 out of the 12 chromosomes, with none found on Chromosome 11. According to the RNA-seq data from pepper CM334, the *CaHsf* members were expressed in at least one tissue among root, stem, leaf, pericarp and placenta. Results of quantitative real-time PCR demonstrated that the *CaHsfs* responded to HS (40 °C for 2 h), except *CaHsfC1* in thermotolerant line R9 leaves, while thermosensitive line B6 showed different response patterns. Many *CaHsfs* were also regulated by salt and osmotic stress, as well as the signal substances Ca^2+^, Put, ABA and MeJA. Further more, CaHsfA2 had the typical characteristics of Hsfs, including being located to the nucleus, having transcriptional activity and responding to continuous HS. Our research not only added a new member to the plant *Hsf* family, but also provided information that could be used in further functional analyses of *CaHsfs* under various abiotic stresses and in elucidating signal transduction pathways in pepper.

## Methods

### Identification and annotation of Hsf family members from *Capsicum annuum*

The conserved amino acid sequence of DBD (Pfam: PF00047) was used as a BLAST query against the pepper genome database PGP (http://peppergenome.snu.ac.kr/, CM334 and Zunla-1 proteins), and the full-length amino acid sequences of the Hsf proteins in *Arabidopsis*, *Vitis vinifera* and *Populus trichocarpa* in the Plant Transcription Factor Database (PTFD) [66] were also used as BLAST query against the PGP and National Center for Biotechnology Information (NCBI). All output genes with default (Limit Expect Value 1e-5) were collected and confirmed by the Pfam (http://pfam.xfam.org/search) and SMART (http://smart.embl-heidelberg.de/). The candidate *Hsf* genes from CM334 and Zunla-1 cultivar were aligned by DNAMAN (Lynnon Biosoft, QC, Canada) for picking out those genes whose sequences were different between the two cultivars. The primers were designed by Primer Premier 5.0 (Premier Biosoft International, CA, USA) (Additional file 6: Table S2) to amplify the different sequences, which were then aligned with the sequences of the same gene from CM334 and Zunla-1 cultivar to confirm the correct sequences. The deduced amino acid sequences were analyzed using Compute pI/MW tool (http://www.expasy.org/tools/pi_tool.html) for computation of the theoretical iso-electric point and protein molecular weight. The classification of candidate Hsf proteins from pepper was performed by Heatster (http://www.cibiv.at/services/hsf/).

### Phylogenetic analysis

Multiple alignments of the N-proximal regions (from the start of the conserved DBD domain to the end of the HR-A/B region) of Hsf proteins from pepper, tomato and *Arabidopsis* were performed by CLUSTALW and the result of alignment was used for the construction of phylogenetic tree [1, 41] using MEGA 5.10 [67]. The parameters for alignment by CLUSTALW were: gap open penalty: 10; gap extension penalty: 0.2; protein weight matrix: Gonnet; residue-specific gap penalties: on; hydrophilic penalties: on; gap separation distance: 0; end gap separation penalty: on; use negative matrix: on; delay divergent cutoff (%): 30. The neighbor joining phylogenetic trees were constructed with pairwise deletion, 1000 bootstraps and a Poisson model.

### Sequence structure analysis and identification of conserved domains in pepper Hsf proteins

Exton/intron organization of the *Hsf* genes in pepper was illustrated with Gene Structure Display Server program (GSDS, http://gsds.cbi.pku.edu.cn/index.php) [68] by alignment of the cDNAs with their corresponding genomic DNA sequences. The MEME program (http://meme-suite.org/tools/meme) was used for identification of conserved motifs, with the following parameters: number of repetitions: any; maximum number of motifs: 25; and the optimum motif widths: 6–200 amino acid residues. The conserved domains annotation was performed using Pfam (http://pfam.xfam.org/search), SMART (http://smart.embl-heidelberg.de/) and Heatster online tools.

### Chromosomal location and gene duplication

Information about the chromosome locations was based on the PGP and the genes were mapped to the chromosomes using MapDraw [69] by identifying their physical chromosome position. Identification of pepper *Hsf* genes duplication was conducted using Plant Genome Duplication Database (PGDD, http://chibba.agtec.uga.edu/duplication/index/locus). Nonsynonymous (Ka) and synonymous (Ks) rates (Ka/Ks) were calculated based on the results of identification of pepper *Hsf* genes duplication, by which the Ks value was converted into divergence time in millions of years based on a rate of 6.1 × 10^−9^ substitutions per site per year, and the divergence time (T) was calculated according to the formula: T = Ks/ (2 × 6.1 × 10^−9^) × 10^−6^ million years ago [44, 45].

### Prediction of protein–protein interaction network

As there were no references for pepper interactome analysis, the interolog from *Arabidopsis* was used for predicting protein–protein interaction network of CaHsf members. First, *Arabidopsis* homologous sequences were searched in INPARANOID (http://inparanoid.sbc.su.se/cgi-bin/index.cgi) [70] based on the sequences of CaHsfs. Then, the edge information file (querynw.sif) of *Arabidopsis* homologs (AtHsfs) were generated via AraNet (http://www.functionalnet.org/aranet/) [71], and mapped to CaHsfs to create an edge information file of CaHsf members. Finally, the protein–protein interaction network of CaHsfs was drawn with Cytoscape_v3.2.1 (National Institute of General Medical Sciences, MD, USA).

### Plant materials, growth conditions and stress treatments

In this study, two pepper lines, thermotolerant line R9 (introduced from the Asia Vegetable Research and Development Center) and thermosensitive line B6 (selected by the pepper research group in the College of Horticulture, Northwest A&F University, Yangling, China) were used. Pepper seedlings were cultivated under a condition of 26/20 °C day/night and a 16/8 h day/night photoperiod in a growth chamber till the 6–8 true leaves period for various treatments. For the HS treatment, the seedlings of B6 and R9 with 6–8 leaves were directly placed in the 40 °C light incubator (GXZ-380C, Jiangnan Instrument Factory, Ningbo, China). The leaves of treated seedlings were collected after 0 and 2 h of HS treatment [37, 38]. For analysis of *CaHsfA2* time-course expression with HS treatment, after being subjected to 40 °C for 0, 0.5, 1, 4, 6 h, and recovered at room temperature (26/20 °C day/night) for 2, 4, 24, 48 h after HS treatment for 6 h, the leaves of B6 and R9 were collected, respectively (Fig. 8a). For other treatments, R9 seedlings were treated with salt stress (300 mM NaCl for 6 h), osmotic stress (5 % mannitol for 6 h), abscisic acid (ABA, 100 μM for 3 h), methyl jasmonate (MeJA , 100 μM for 6 h), CaCl_2_ (15 mM for 6 h ) and putrescine (Put, 1.5 mM for 6 h). MeJA was dissolved in 10 % ethanol, other substances were dissolved in water, and control seedlings were treated with 10 % ethanol (for MeJA treatment) or water (for NaCl, mannitol, CaCl_2_, Put and ABA treatment). The roots and stems were collected from seedlings treated with NaCl and mannitol, and the leaves were sampled for other treatments. The samples were frozen in liquid nitrogen and stored at −80 °C for RNA extraction. Each treatment was conducted with three biological replicates, and samples from five seedlings were gathered for each replicate.

### RNA extraction and quantitative real-time PCR analysis

Total RNA were isolated according to the instruction of Total RNA kit (BioTeke, Beijing, China), and the cDNA was synthesized according to the manufacturer’s instructions (Takara, Dalian, China) and was diluted to 50 ng/μL with ddH_2_O. For quantitative real-time PCR (qRT-PCR) assay, primer pairs (Additional file 7: Table S3) for pepper *Hsf* genes were designed at the C-terminal domain by Primer Premier 5.0, and their specificity was checked by NCBI Primer BLAST (http://www.ncbi.nlm.nih.gov/tools/primer-blast/index.cgi?LINK_LOC=BlastHome). Ubiquitin binding protein gene *UBI-3* from pepper was used as the reference gene [72]. The qRT-PCR reactions were carried out on the iQ5.0 Bio-Rad iCycler thermocycler (Bio-Rad, Hercules, CA, USA) using SYBR Green Supermix (Takara, Dalian, China). The 20 μL reaction system contained 10 μL SYBR Green Supermix (2×), 2 μL cDNA template (50 ng/μL), 0.8 μL of each primer (10 μM) and 6.4 μL ddH_2_O. The qRT-PCR reaction systems were as follows: pre-denaturation at 95 °C for 1 min, followed by 40 cycles of denaturation at 95 °C for 10 s, annealing at 56 °C for 30 s, extension at 72 °C for 30 s. The fluorescent signal was measured at the end of each cycle, and the melting curve analysis was performed with heating the PCR product from 56 °C to 95 °C for verifying the specificity of the primers. Three independent biological replicates were carried out and qPCR of each replicate was performed in triplicate. The relative expression levels of pepper *Hsf* genes were calculated as -ΔΔCT method [73].

### Pepper *Hsf* genes tissue-specific expression analysis

Based on the pepper genes against RNA-seq data of each tissue [42], we chose the data of 17 tissues and developmental stages including root, stem, leaf, pericarp and placenta at 6, 16, 25 days post-anthesis (PC-6DPA, −16DPA and -25DPA, PL-6DPA, −16DPA and -25DPA ), pericarp and placenta at mature green (PC-MG, PL-MG) and at breaker (PC-B, PL-B), pericarp and placenta at 5 and 10 days post-breaker (PC-B5 and -B10, PL-B5 and -B10) from CM334 for the *CaHsf* genes tissue- and stage-specific expression analysis. The data of the 22 pepper *Hsf* genes (excluding *CaHsfA1e*, *B3b* and *B4* whose date are absent) were used for creating a heat map using HemI (The Cuckoo Workgroup, Wuhan, China).

### Subcellular localization of CaHsfA2

Based on our previous study [38], the ORF of *CaHsfA2* without the termination codon was prepared by PCR using cDNA from R9 leaves treated with 40 °C for 2 h as the template and a primer pair (forward primer 5'-GCTCTAGATCCATCTTAATTGTATTTAGCGAC-3' and reverse primer 5'-CGGGGTACCAAGGAAACCAAGTTGATCTACAAG-3'). The underlined nucleotides contained the *Xba*I and *Kpn*I restriction site, respectively. The PCR-amplified *CaHsfA2* fragment was fused to the pBI221 expression vector and the CaMV35S::GFP (pBI221) vector without *CaHsfA2* was used as a control. For transient expression analysis, 5 μg of CaMV35S::CaHsfA2-GFP plasmid or CaMV35S::GFP plasmid was introduced into the onion (*Allium cepa*) epidermal cells using a Bio-Rad He/1000 particle delivery system (Bio-Rad, Hercules, CA, USA). Bombarded cells were incubated at 28 °C for 24 h on 1× MS (Murashige and Skoog) agar medium, and then green fluorescent protein (GFP) fluorescence was observed using an A1R confocal-laser scanning microscope (Nikon, Tokyo, Japan).

### Analysis of transcriptional activation of CaHsfA2 in yeast cells

Transcriptional activation of CaHsfA2 assay was performed in the yeast strain AH109 with *LacZ* and *His* reporter genes. The complete coding sequence of *CaHsfA2* was amplified by PCR using primers (forward primer 5'-TCCATCTTAATTGTATTTAGCGAC-3' and reverse primer 5'-AGGGAATGATAGAGTCGTGGG-3'). The PCR products were cloned into pMD19-T vector (Takara, Dalian, China), and the confirmed fragment (*Sma*I site of pMD19-T vector linked with the 5' untranslated region of *CaHsfA2* and *Pst*I site linked with the 3' untranslated region) was cut with restriction enzymes *Sma*I and *Pst*I from pMD19-T vector, and then linked into *Sma*I/*Pst*I sites in the pGBKT7 vector to create pGBKT7-CaHsfA2. The constructs of pGBKT7-CaHsfA2 and the negative control pGBKT7 were cloned into AH109 yeast strain, respectively, according to the manufacturer’s protocol (Clontech, Palo Alto, CA, USA). Transformed strains were confirmed by PCR and sequencing and then plated on SD/Trp- or SD/Trp-Ade-His- plates. Transcription activation was evaluated according to the growth status of yeast cells after incubating plates at 30 °C for 3 d with 5-bromo-4-chloro-3-indoxyl-α-D-galacto-pyranoside (X-α-gal).

### Availability of supporting data

The phylogenetic data in this publication are available for download at TreeBase with accession number 17530 (http://purl.org/phylo/treebase/phylows/study/TB2:S17530). The RNA-seq data were available from genome of pepper cultivar CM334 (http://www.nature.com/ng/journal/v46/n3/full/ng.2877.html#supplementary-information) [42].

## Additional files

Additional file 1: Fig. S1. Multiple sequence alignment of the DBD domains of 25 members of the Hsf protein family in pepper. The definition of the Hsf names corresponded to the order of alignment. The multiple alignment result clearly shows the highly conserved DBD domains among pepper *Hsf* genes. The 3D structure and the secondary elements (α1-β1-β2-α2-T-α3-β3-β4) are shown above the alignment. T: turn of helix-turn-helix motif. Additional file 2: Fig. S2. Phylogenetic analysis (**A**) and exon/intron organizations (**B**) of pepper *Hsf* genes. Numbers above or below branches in (**A**) indicates bootstrap values. Differently colored lines shows genes in each subclass. Numbers 0, 1 and 2 in (**B**) represent introns in phases 0, 1 and 2, respectively. Additional file 3: Fig. S3. Chromosomal mapping of pepper *Hsf* gene family. Chromosomal mapping was based on the physical position (Mb) in 11 out of 12 pepper chromosomes. The chromosome numbers are indicated at the top of each chromosome. Chromosomal positions of the pepper *Hsf* genes are indicated by gene names. Blue and gray dotted lines connect the *CaHsf* genes present duplicate chromosomal segments. Additional file 4: Table S1. Divergence between *CaHsf* genes pairs in pepper. Additional file 5: Fig. S4. The predicted protein–protein interaction network of CaHsfs. Additional file 6: Table S2. Primers for amplifying the different sequences between CM334 and Zunla-1 genome among *CaHsf* members. Additional file 7: Table S3. Primer sequences used for quantitative real-time PCR analysis.

## Abbreviations

Hsfs: Heat shock factors
qRT-PCR: Quantitative real-time PCR
HS: Heat stress
Hsps: Heat shock proteins
HSEs: Heat shock elements
DBD: DNA-binding domain
OD: Oligimerisation domain
AA: Amino acid
NLS: Nuclear localisation signal
NES: Nuclear export signal
RD: Repressor domain
HMM: Hidden Markov Model
CBK: Calcium/calmodulin-binding protein kinase
PGDD: Plant Genome Duplication Database
GFP: Green fluorescent protein
PTFD: Plant Transcription Factor Database
NCBI: National Center for Biotechnology Information
Put: Putrescine
ABA: Abscisic acid
MeJA: Methyl jasmonate
X-α-gal: 5-bromo-4-chloro −3-indoxyl-α-D-galacto-pyranoside
