# Supplementary figures and images for: Genome-wide analysis, expression profile of heat shock factor gene family (CaHsfs) and characterisation of CaHsfA2 in pepper (Capsicum annuum L.)
Source: BMC Plant Biol. 2015 Jun 19;15:151. doi: 10.1186/s12870-015-0512-7 (PMC4472255; doi:10.1186/s12870-015-0512-7)

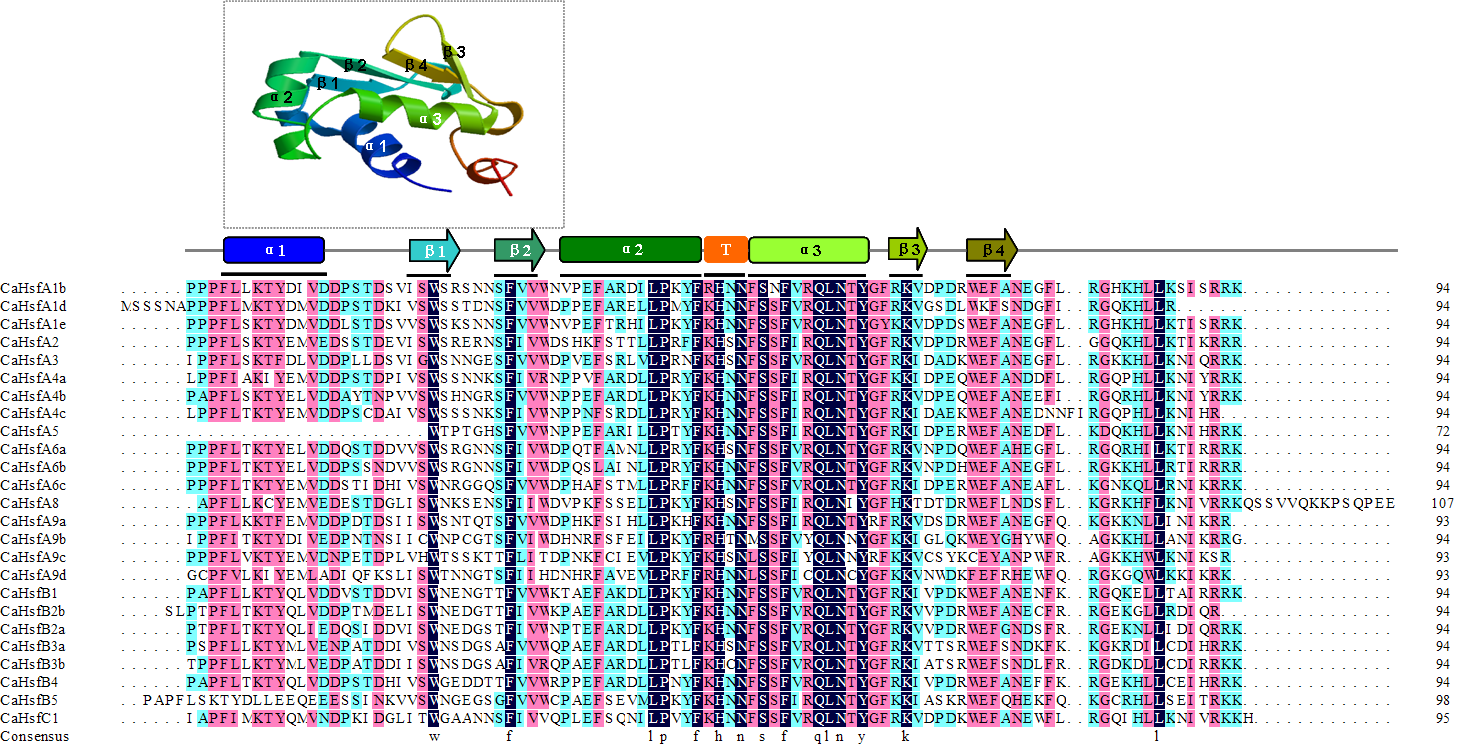

Supplement: Additional file 1: Fig. S1. — Multiple sequence alignment of the DBD domains of 25 members of the Hsf protein family in pepper. The definition of the Hsf names corresponded to the order of alignment. The multiple alignment result clearly shows the highly conserved DBD domains among pepper Hsf genes. The 3D structure and the secondary elements (α1-β1-β2-α2-T-α3-β3-β4) are shown above the alignment. T: turn of helix-turn-helix motif. [file 12870_2015_512_MOESM1_ESM.png]

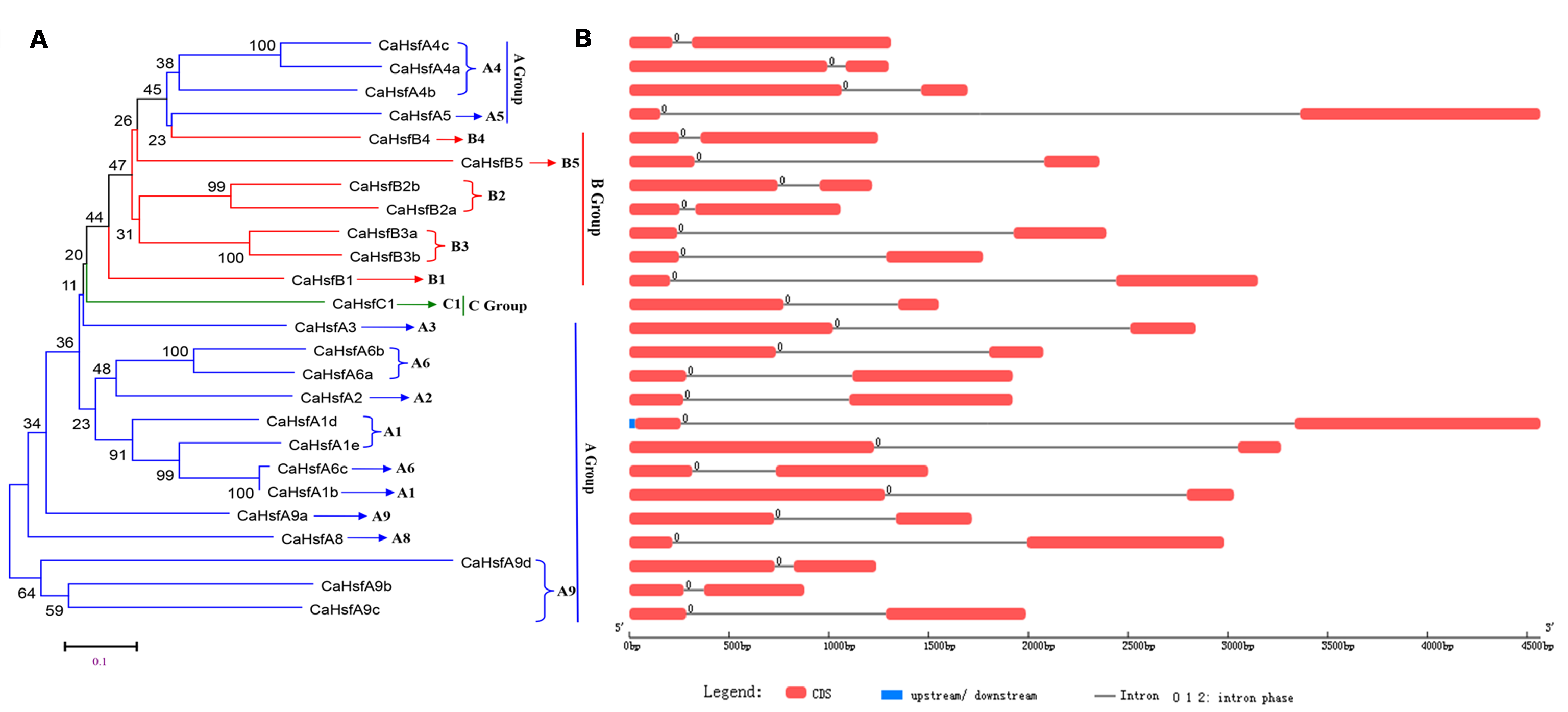

Supplement: Additional file 2: Fig. S2. — Phylogenetic analysis (A) and exon/intron organizations (B) of pepper Hsf genes. Numbers above or below branches in (A) indicates bootstrap values. Differently colored lines shows genes in each subclass. Numbers 0, 1 and 2 in (B) represent introns in phases 0, 1 and 2, respectively. [file 12870_2015_512_MOESM2_ESM.png]

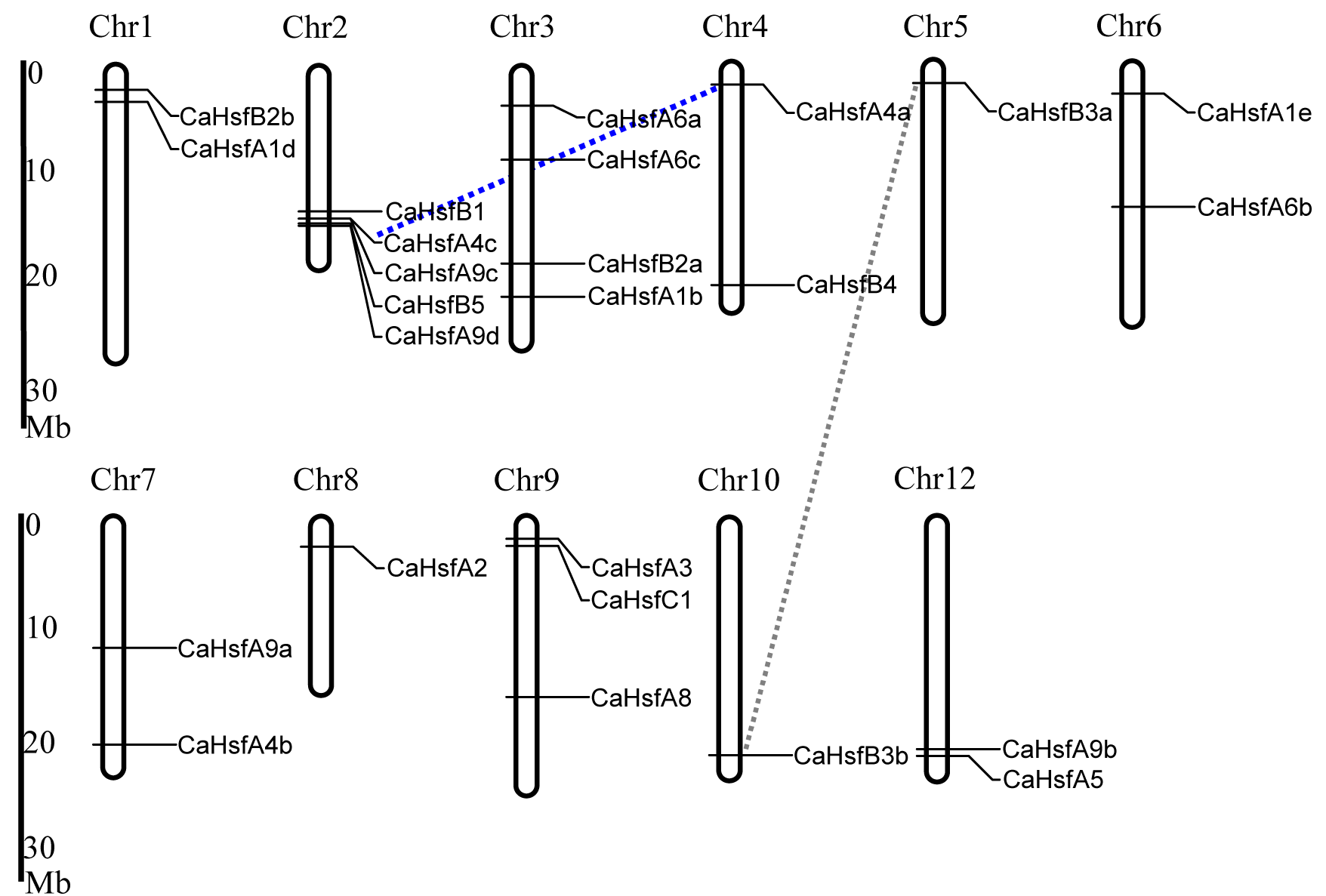

Supplement: Additional file 3: Fig. S3. — Chromosomal mapping of pepper Hsf gene family. Chromosomal mapping was based on the physical position (Mb) in 11 out of 12 pepper chromosomes. The chromosome numbers are indicated at the top of each chromosome. Chromosomal positions of the pepper Hsf genes are indicated by gene names. Blue and gray dotted lines connect the CaHsf genes present duplicate chromosomal segments. [file 12870_2015_512_MOESM3_ESM.png]

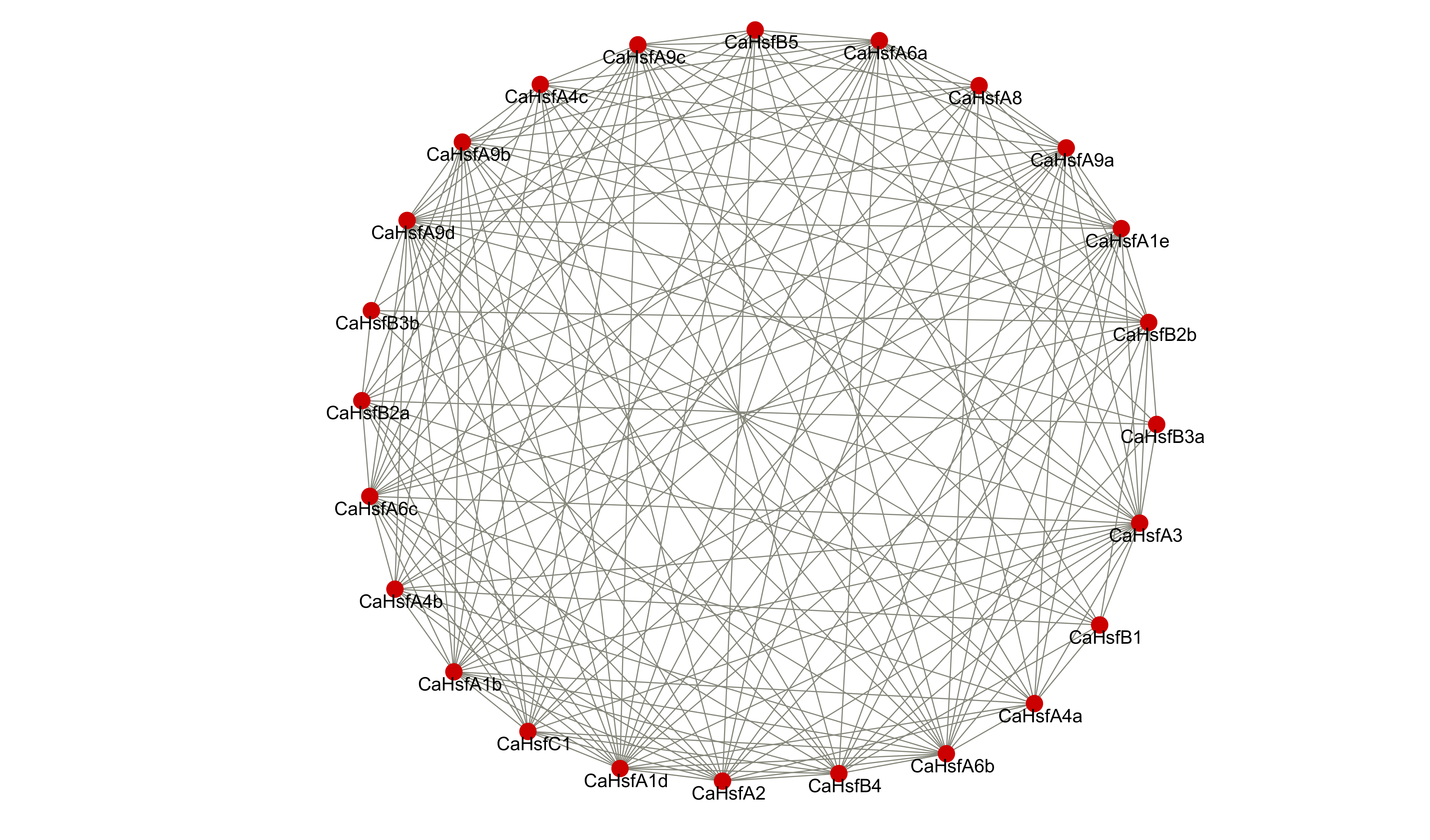

Supplement: Additional file 5: Fig. S4. — The predicted protein–protein interaction network of CaHsfs. [file 12870_2015_512_MOESM5_ESM.png]
