# Supplementary material for: Genome-wide analysis, expression profile of heat shock factor gene family (CaHsfs) and characterisation of CaHsfA2 in pepper (Capsicum annuum L.)
Source: BMC Plant Biol. 2015 Jun 19;15:151. doi: 10.1186/s12870-015-0512-7 (PMC4472255; doi:10.1186/s12870-015-0512-7)
Supplement: Additional file 4: Table S1. — Divergence between CaHsf genes pairs in pepper. [file 12870_2015_512_MOESM4_ESM.doc]

**Table S1** Divergence between *CaHsf* genes pairs in pepper.

| **Paralogous pairs** | **Ka** | **Ks** | **Ka/Ks** | **Duplication Date(MY)** | **Duplicate type** |
| --- | --- | --- | --- | --- | --- |
| CaHsfA4a-CaHsfA4c | 0.20 | 0.56 | 0.357 | 45.9 | Segmental |
| CaHsfB3a-CaHsfB3b | 0.17 | 0.87 | 0.195 | 71.31 | Segmental |

For each gene pair, the Ks value was translated into divergence time in millions of years based on a rate of 6.1×10^-9^ substitutions per site per year. The divergence time (T) was calculated as T=Ks/(2×6.1×10^-9^)×10^-6^ Mya.
