# Supplementary material for: Genome-wide analysis, expression profile of heat shock factor gene family (CaHsfs) and characterisation of CaHsfA2 in pepper (Capsicum annuum L.)
Source: BMC Plant Biol. 2015 Jun 19;15:151. doi: 10.1186/s12870-015-0512-7 (PMC4472255; doi:10.1186/s12870-015-0512-7)
Supplement: Additional file 6: Table S2. — Primers for amplifying the different sequences between CM334 and Zunla-1 genome among CaHsf members. [file 12870_2015_512_MOESM6_ESM.doc]

***Table S2 Primer sequences were used for identifying the difference sequences between CM334 and Zunla-1 genome among CaHsfs***.

| **Gene name** | Forward primer(5′→3′) | Reverse primer(5′→3′) |
| --- | --- | --- |
| CaHsfA1d | ATGGGTTCTGCTTCAATGG | TCATACTTTTTTACTGTTTGATGTTAG |
| CaHsfA1e | ATGGAAGGAGTTAATGAGATAGG | TTAAGTGAGATGACATATACGCTT |
| CaHsfA4a | ATGGATGAAGCTCCTTGCAG | TTACGATCTGTCTGCTGGAGT |
| CaHsfA4b | ATGGTAAGCATAGTCATGGAAAAT | TCAACTTCCTGTTGTTGCTGG |
| CaHsfA5 | ATGGACGTAATTTCTCCAGCG | TCAAAGGGTAAGATGTTGTACCTT |
| CaHsfA6b | ATGGATCATTTTGGTAATTCAAT | TCATTCAGACAAAATATCTACATG |
| CaHsfA6c | ATGAATAATAATCCATTGTATTCG | CTAAGGGCTCGAATCCATG |
| CaHsfA9d | ATGGAAATAGATAGAGGAAAAGG | TTATGACATTATGACTAGCAGATAAGT |
| CaHsfB2b | ATGGTGCCGTCGTCGGTT | TCATATCCATTGTTGATCATCATG |
| CaHsfB3a | ATGGAGGTATTGGAAGAAGTTC | TTATTTGCATAATTGAGAGATAAAAATAT |
| CaHsfB3b | ATGGGGAATTTAGAGGGTGATC | TTATTTGCATAATTGAGATAGAAAAACAC |
| CaHsfB4 | ATGGCTTTGATGTTGGACAATT | TTAAGATGATGAAGAAGGGGGC |
| CaHsfB5 | ATGGCCACAGATGCTATTCC | CTAGTTTTTATTATGTGGACTTTCC |
| CaHsfC1 | ATGGAGGCTAACAATATAATCG | CTAAAATCCACCACCTAATAAAG |
