# Supplementary material for: Genome-wide analysis, expression profile of heat shock factor gene family (CaHsfs) and characterisation of CaHsfA2 in pepper (Capsicum annuum L.)
Source: BMC Plant Biol. 2015 Jun 19;15:151. doi: 10.1186/s12870-015-0512-7 (PMC4472255; doi:10.1186/s12870-015-0512-7)
Supplement: Additional file 7: Table S3. — Primer sequences used for quantitative real-time PCR analysis. [file 12870_2015_512_MOESM7_ESM.doc]

**Table S3 Primer sequences were used for quantitative real-time PCR analysis.**

| **Gene name** | Forward primer(5′→3′) | Reverse primer(5′→3′) |
| --- | --- | --- |
| CaHsfA1b | GCTGACTTTAAGATGTCCGAGAC | GCTTTAGTTTATCCCAATCACTC |
| CaHsfA1d | GAGCCCTCACAGTTCCAAG | AAGAATCAATGCCCGTCTG |
| CaHsfA1e | TGACTAATCGTTCTCGGTCTT | TGACTAATCGTTCTCGGTCTT |
| CaHsfA2 | GTAGCATCAGTAGCCACAGC | CAAGCAACTCTTCCCAAATA |
| CaHsfA3 | CGAAAGTATGATGAAAGAAGAGG | ATAGTTGCCAAGACCACCC |
| CaHsfA4a | CAATTCCTGACTGAGAACCCTG | TCCGTTTCCGCCTTTACAT |
| CaHsfA4b | TGTGATAGCAGGTTAGTTGAA | TAAGATGTCCCATTCGTTC |
| CaHsfA4c | GCAAACCCTACTCCTGATG | GCCAGGCTATTTACACTCTT |
| CaHsfA5 | GTCGGTGGACAACCATAGC | CTGAAACAGCAGGTGACAAT |
| CaHsfA6a | CAAGGACAAAGTGGGAACA | AAATCCAAGATGATGAGCC |
| CaHsfA6b | GGACCTCCACCATTTCTTA | TTGTTACCTCTACTCCACGA |
| CaHsfA6c | AAGGCTCTAGTAGCAGTACATTAC | CTCTTCACCGTCTTCATTTT |
| CaHsfA8 | ACTCAGAAACCGATGGAGC | GGCAAAGTAAGAGGGACACTA |
| CaHsfA9a | CCCTCTTTGTTCAGCATCT | AGCGTTAGTCAACCTCCTC |
| CaHsfA9b | CGTGGTATGAAGCGTACTGA | GTGCTCGTTTAATTGCAGAA |
| CaHsfA9c | GGATGGGATTGGACAGACA | TAAGCCTCCGCCTCGTCAT |
| CaHsfA9d | TTCTTGGAGTCGGAGGAGC | TGTAATGGTTCAAGGTGGC |
| CaHsfB1 | AGGTTGCACCCGATATGAT | AACTCTTCAAGGCTTGTCCC |
| CaHsfB2a | ATTATTCGGGTTCTCGATAGG | ACTCCAGGTTGCTGTAGGC |
| CaHsfB2b | TGAATCGGTTACGAGGTTTATG | TGATCCTGGCACCTTCCTT |
| CaHsfB3a | CGACCGACGACATCGTTTC | TTGTCATTGCTGAACTCCC |
| CaHsfB3b | CATCAACACCACCATTCTT | CATAGTAAATCCTTGTCTCCC |
| CaHsfB4 | AATGTTCTTATGGGCAACT | TGTACTTCTATTATCACCTCCA |
| CaHsfB5 | AAAGTTCCAGAAAGGGTGC | TCCATACATTGAGTGAGGC |
| CaHsfC1 | GTGTAAAGTTGTTGATGACCCTG | GACGACGGCGAAGACTGAC |
| UBI-3 | TGTCCATCTGCTCTCTGTTG | CACCCCAAGCACAATAAGAC |

Primers were designed at the C-terminal domain by Primer Premier 5.0, and their specificity was checked by NCBI Primer BLAST. Ubiquitin binding protein gene *UBI-3* from pepper was used as the reference gene.
